# A New Jasmine Virus C Isolate Identified by Nanopore Sequencing Is Associated to Yellow Mosaic Symptoms of *Jasminum officinale* in Italy

**DOI:** 10.3390/plants11030309

**Published:** 2022-01-24

**Authors:** Serafina Serena Amoia, Angelantonio Minafra, Vittorio Nicoloso, Giuliana Loconsole, Michela Chiumenti

**Affiliations:** Institute for Sustainable Plant Protection—National Research Council, 70126 Bari, Italy; serena.amoia@ipsp.cnr.it (S.S.A.); angelantonio.minafra@ipsp.cnr.it (A.M.); vittorio.nicoloso@ipsp.cnr.it (V.N.); giuliana.loconsole@ipsp.cnr.it (G.L.)

**Keywords:** jasmine, virus, yellow mosaic, high-throughput sequencing, virus detection, *Carlavirus*

## Abstract

Some plants of *Jasminum officinale* were selected in a nursery for investigation of sanitary status of candidate mother plants before vegetative propagation. The presence of yellow spots and leaf discoloration symptoms pushed for a generic diagnosis through deep sequencing to discover systemic pathogens. Either dsRNA or total RNA were extracted and used in nanopore and Illumina platform for cDNA-PCR, direct RNA and total RNA rRNA-depleted sequencing. A few single reads obtained by nanopore technology or assembled contigs gave unequivocal annotation for the only presence of a jasmine virus C (JaVC, a putative member of genus *Carlavirus*) isolate. The full-length genome of this isolate was reconstructed, spanning 8490 nucleotides (nt). This isolate shared 90.9% similarity with coat protein sequences and 84% with the entire ORF1 polyprotein, with the other two available JaVC full genomes, isolated from infections in *J. sambac* in Taiwan and China. The overall nucleotide identity shared by the newly discovered Italian isolate with the Chinese JaVC full genomes was 76.14% (Taiwan) and 75.60% (Fujian). The application of quick nanopore sequencing for virus discovery was assessed. The identification of the virus in a new ornamental host species, largely used in gardening, creates a concern for the potential virus spread and need of testing for production of clean vegetative material.

## 1. Introduction

Symptoms due to the systemic infection of viruses and virus-like pathogens in vegetatively propagated ornamental plants have been widely described in the past [1,2] even if they could have been largely underestimated in case of latency or mild symptoms of the infection [3,4]. Apart from the wide number of conventional studies performed in the last decades on specific ornamental host–virus associations, the application of high-throughput sequencing (HTS) in plant virology since 2009 [5] demonstrated to be a key technical outbreak. The quick molecular identification and characterization by HTS of viruses and viroids has been developed by using all the nucleic acid templates suitable to the scope (small RNAs, enriched genomic targets of pathogens, total RNA or DNA) and libraries run on different platforms (like Illumina or nanopore sequencing; [6,7,8]). This technique allowed the discovery of new viruses infecting several hosts, including ornamental and cultivated plants, whether the infections were able to express symptoms or not, as well as the detection of already described viruses in new hosts [9,10]. The critical role and potential danger played by these virus-infected ornamental hosts as inoculum reservoir and/or alternate hosts, and the importance of timely and accurate pathogen detection and disease management, have been also posed [3,11,12,13]. An under-evaluated risk is that increased international trades of ornamental plants facilitate the introduction of new pathogens, which in turn may cause disease outbreaks through unaware transmission to new hosts or crops, endangering new ecological and agricultural areas previously free from the specific alien pathogen.

Jasmine is an important ornamental shrub grown worldwide for garden decoration or the essential oil extraction industry. In *Jasminum* spp., since 1971 [14], a diverse array of viruses has been identified and reported. These agents are variously linked to symptom evidence. A recent review [15] lists 13 different viruses belonging to several families affecting *Jasminum*. One of the most relevant symptoms is the yellow spot or mosaic. In early observations, flexuous virus particles were observed in *J. sambac* plants which showed viral symptoms in the Philippines and Italy [16,17]. Bellardi and Bertaccini [17], testing extracts from those infected plants, found partial cross-reactions to antisera against two viruses, Narcissus latent virus (NLV, genus *Macluravirus*) and shallot latent virus (SLV, genus *Carlavirus*). A later report associated the potyvirus jasmine virus T (JaVT) to a similar syndrome—mosaic and mild mottling—in the same host in Taiwan [18]. From *J. sambac* sources, in Taiwan too, a new carlavirus, jasmine virus C (JaVC), was identified in 2012, and its full genome was sequenced [19]. This virus was apparently not transmissible on herbaceous test hosts, but always associated in the original jasmine host to pronounced yellow mosaic and not to milder symptoms. Recently, a sequencing project was performed on JaVC isolates infecting *J. sambac* in different Chinese locations. The sequences of an amplified fragment of 1275 nt, from the 3’ end of the JaVC genome of those isolates, are reported in databases.

However, the etiology of this syndrome in jasmine appears still unresolved to date. An uncharacterized virus (Jasminum chlorotic ringspot virus, JCRSV) was also described in 1972 in India [20] as putatively transmissible by whiteflies. No comparison was made with the Italian isolate of carlavirus [17], but now it is known that at least two carlaviruses (cowpea mild mottle virus and melon yellowing-associated virus) are whitefly-transmitted. Dey et al. claimed that two other distinct pelarspoviruses (jasmine virus H, JaVH, and jasmine mosaic-associated virus, JMaV) were co-infecting *Jasminum* species (*J. multiflorum* and *J. nitidum*) showing yellow ringspots at different extent in several locations in the USA and Hawaii [21].

We encountered, in the frame of a Regional Project aimed at surveying and enhancing the phytosanitary quality of some ornamental species during the propagation pipeline, yellow spots and interveinal mosaic symptoms on some clones of *J. officinale* from a nursery in Apulia (Italy) and our work, based on HTS application also through preliminary in-house nanopore fast sequencing, aimed to clarify if and which viral entities could be associated to these symptoms.

## 2. Results

### 2.1. Sequencing Results

#### 2.1.1. Nanopore

One retro-transcribed dsRNA library and one direct RNA library were prepared for Oxford Nanopore Technology (ONT) sequencing from the *J. officinale* mixed sample (done by pooling tissues from three different accessions). After 7 h of sequencing, a total of 425,637 and 328,990 raw reads were obtained from dsRNA and direct RNA libraries, respectively. Quality controls with the Epi2me tool generated a total of 347,277 and 250,448 of quality passed reads, with a yield of 81.59 and 76.13% of good quality (Phred score ≥ 8) reads for dsRNA and direct RNA, respectively (Table 1).

When all quality filtered reads were annotated, using either BlastN vs. nt database or BlastX against customized viral databases, searching for virus and viroid-similar genomes, the annotation of retrotranscribed dsRNAs recognized as carlavirus-like two contigs with BlastN and seven additional contigs using BlastX, respectively. All nine carlavirus-like reads ranged from 113 to 971 nt length and showed sequence homology to that of jasmine virus C (JaVC). Seven reads mapped in the replicase gene, one in the putative nucleic acid-binding protein and one in the coat protein. No other viruses or viroids were identified by blast annotation.

BlastN annotation of direct RNA sequencing allowed the identification of two reads of 1545 and 5009 nt length with high homology to JaVC isolate A-31 (Accession number, acc. nr. KX364696). The 1545 nt long contig, positively stranded, aligned to JaVC A-31 from position 7047 to 8320, with 76% of sequence identity, whereas the 5009 nt length read, positively stranded too, matched to JaVC A-31 reference sequence from position 3548 to 6256. BlastX annotation of the same dataset of reads allowed the identification of 14 additional reads with sequence homology to JaVC and other carlaviruses and lengths ranging from 423 to 1124 nt. No other known or putative novel viruses were identified with BlastN or BlastX annotations of direct RNA reads.

#### 2.1.2. Illumina

Illumina sequencing of the total RNA from *J. officinale* library yielded a total of 18,878,273 paired-end reads. After the quality filtering and cleaning-up steps, a total of 18,784,933 pairs, corresponding to 99.5% of total raw reads, were obtained. High-quality reads were de novo assembled using the SPAdes algorithm, producing 520,748 contigs with lengths ranging from 122 to 40,186 nucleotides. Blast annotation of the obtained contigs allowed the identification of a single fragment of 8344 nt in length with the highest homology to jasmine virus C isolate A-31, with negative orientation, query coverage of 99% and percentage identity of 76%. No other known or putative novel viruses were identified neither with BlastN or BlastX annotations.

### 2.2. Genome Characterization of the Carlavirus Isolate

Since the BLAST result of the contigs query from *J. officinale* (at first from nanopore libraries and after from Illumina one) returned viral sequences only matching with isolates of jasmine virus C and some other related carlaviruses, the reconstruction of the full-length virus genome was pursued. The 5’ terminus obtained by the Illumina assembled contig perfectly matched with both the homologous sequences from the full-length genomes of JaVC (isolates Fujian, acc. nr. MH231174, and A-31), and no need to run a 5’ RACE experiment was envisaged. Instead, the genomic sequence at 3’ portion stopped in a putative coding region, shortly ahead of the untranslated region. The sequenced clones from the amplicon produced by J3’for and the reverse oligo-dT-anchor primers (Supplementary Materials Table S1) gave an invariable size of 243bp up to the polyA stretch.

**Figure 1:**
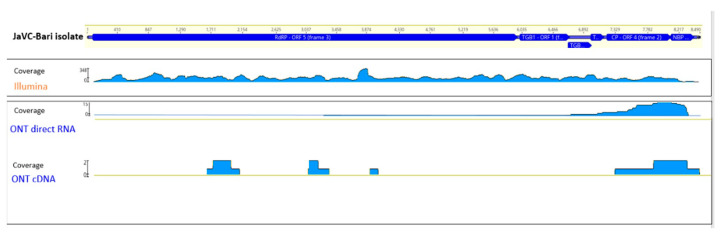
Genomic organization of JaVC. Distribution and respective coverage of the reads obtained from the three techniques along the genome of JaVC-Bari isolate.

After the fusion of this last terminal portion, the obtained full-length genome had a final size of 8490 nt (Figure 1; sequence deposited to GenBank, acc. nr. OL828237). The 5’ noncoding region consisted of 71nt. Open Reading Frame (ORF1) (start 72, stop 5972; 5901 nt, 1966 amino acids, (aa)) polypeptide contained seven expected domains described in the *Carlavirus* genus for the replicase gene (see Supplementary Materials Figure S1).

A first intergenic region of 31 nt separated ORF1 from the triple gene block (TGB), a set of three overlapping small ORFs with proteins having coordinated functions dedicated to virus movement. ORF2 (TGB1) contains a conserved motif of G-GKSSL, which has been found in various ATP- and GTP-binding domains [22]. ORF3 (TGB2) is a 12kDa protein with two transmembrane domains and the central conserved signature GD--H-LPHGG-Y-DGTK. It was recognized by InterPro as containing plant viral movement protein motifs (pfam 01307). The smallest gene of the set (ORF4; TGB3), a 7kDa protein, showing a single transmembrane helix configuration, features the conserved motif CV--ITGES [23] (Supplementary Materials Figure S1).

A second small intergenic region of 42 nt was present ahead of the ORF5. In the ORF5 gene (296 aa), InterPro identified two highly conserved domains of the flexivirus coat protein (CP) (a carlavirus coat N signal, pos. 52–103; and a flexivirus CP, pos. 113–252). A “disorder-domain” [24] and a coil oligopeptide were also indicated at positions 1–44 and 46–66, respectively, ahead of the latter domains. Finally, a 3’ terminal ORF (ORF6; 105 aa) had the starting ATG overlapping with the stop codon of the CP gene. Notably, 3 amino acid positions in this protein were divergent if compared with the other 10 available JaVC sequences in this region, all derived from Eastern Asia isolates (Supplementary Materials Figure S2). Neutral amino acids (isoleucine-34 and threonine-68 and -72) substituted conserved residues in the alignment: either neutral (alanine/valine, pos. 34; serine/cysteine, pos. 68) or an invariant basic arginine (pos. 72).

### 2.3. Genetic Variability and Phylogeny

The discovery of a different JaVC isolate in a new *Jasminum* species required a comparison of genetic variability of the isolate among the few other entries of the same virus sequences in database, as well as among closely related members of *Carlavirus* genus. A preliminary analysis of the full-length JaVC-Bari genome, run as BlastN (somewhat similar nucleotide sequences) retrieved a wide array of similarity in the genus, ranging from the highest with Hippeastrum latent virus (HLV) (identity 68%; query coverage 57%) to the lowest for cowpea mild mottle virus (CPPMV) (id. 71%, but on a query coverage of 22% only). The megablast (highly similar nucleotide sequences) search gave an obvious similarity with carlaviruses, too. The first hit after JaVC isolates was Chrysanthemum virus B (CVB), 72.7% identity on 10% of the JaVC genome (in the region 5018–5921, corresponding to the core RNA-dependent RNA polymerase (RdRp)). However, 71% of identity, on a fragment spanning 9% of the JaVC genome in the same replicase region, was also observed with several isolates of the foveavirus grapevine virus T [25] (not shown).

**Figure 2:**
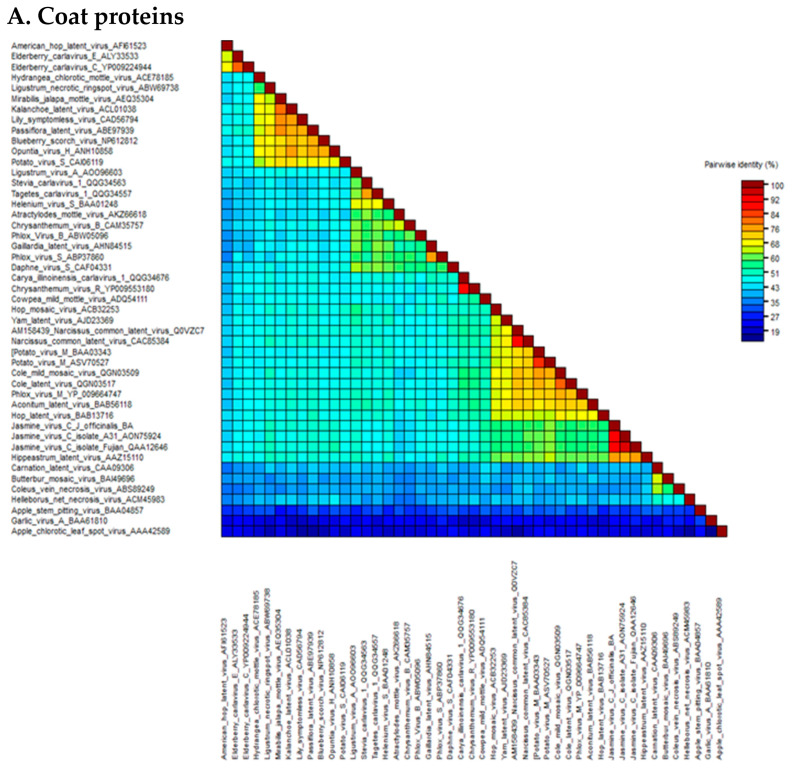

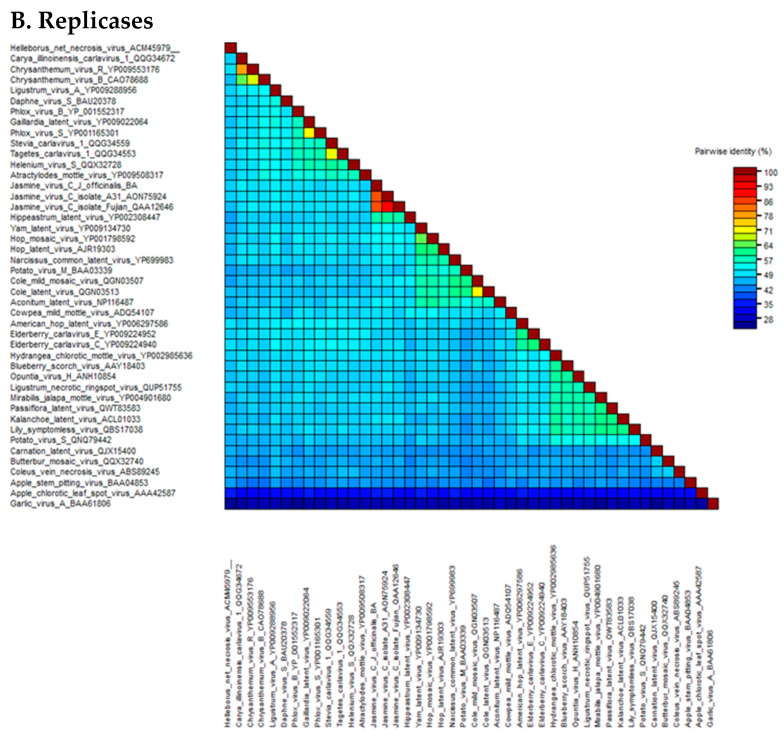
Heat map graphics of pairwise identity matrix analysis for coat proteins (**A**) and ORF1 polyprotein (**B**) of the JaVC isolates compared to other carlaviruses and outgroup flexivirids homolog proteins. GenBank accession numbers are by the virus name (refer also to Supplementary Materials Table S2).

The pairwise matrix comparison of JaVC amino acidic sequences of the whole replicase and coat proteins was performed among the homologous sequences of carlaviruses and three outgroup flexivirid viruses (apple stem pitting virus, ASPV; garlic virus A, GaVA and apple chlorotic leaf spot virus, ACLSV) (all the viral protein sequences used in the study are listed in Supplementary Materials Table S2). In the CP gene, the range of similarity went from 41.7 to 42.7% for Helleborus net necrosis virus and American hop virus, up to 60.7% for potato virus M and 75.7% for Hippeastrum latent virus (Figure 2A; Supplementary Materials Table S3). For the replicase, calculated values were from a minimum of 43.9% for Coleus vein necrosis virus and carnation latent virus to 51.4, 51.6 and 57.4% for hop mosaic virus, yam latent virus and Hippeastrum latent virus, respectively (Figure 2B; Supplementary Materials Table S4).

A higher similarity resulted when an inter-isolate comparison was performed among JaVC-Bari and the other two JaVC isolates (derived from *J. sambac* infections in Taiwan and China), for which a full-length genome is available (A-31 and Fujian). Both coat proteins shared a 90.9% similarity with JaVC CP (between them it was 94.6%), while for the entire ORF1 polyprotein, they showed 84% similarity with JaVC-Bari and 92.1% between them. When the full-length genome comparison was tested at the nucleotide level by BLASTn, full coverage was achieved with the other two JaVC isolates with an identity of 76.14% (A-31 Taiwan) and 75.60% (Fujian), respectively.

Furthermore, the availability of a high coverage for the mapping on the assembled genome (average of 8.81x) obtained from the Illumina sequencing allowed the annotation of intra-isolate JaVC-Bari variability. At least 32 single-nucleotide polymorphisms (SNPs) could be listed, with a prevalence of transitions (27 out of 32 SNPs), having a variant frequency from 10.0 (*p*-value 2.1 × 10^−12^) to 54.5% (*p*-value 2.1 × 10^−150^). Only seven polymorphisms could result in a potential change of amino acid residue, while all the others (located mainly in the ORF1 coding sequence) were related to synonymous mutations (Supplementary Materials Table S5).

**Figure 3:**
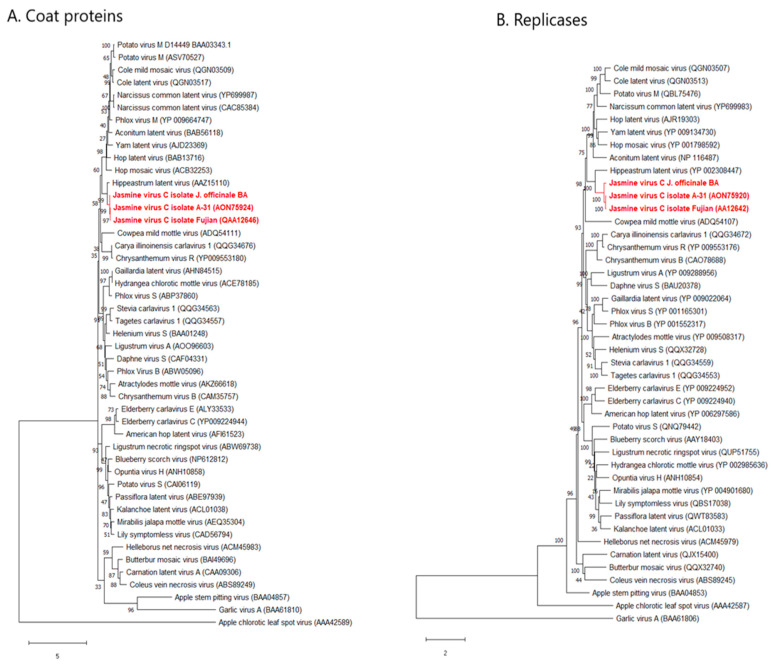
Maximum likelihood phylogenetic analysis of coat proteins (**A**) and ORF1 polyprotein (**B**) of the JaVC isolates (in red) versus other carlaviruses and outgroup flexivirids homolog proteins. GenBank accession numbers are on the tips (refer also to Supplementary Materials Table S2). Distance bar unit is under the dendrograms.

On the same amino acidic sequences analyzed in the pairwise comparison, maximum likelihood phylogenetic trees were constructed (Figure 3A,B). The coat proteins of JaVC isolates were closely related to Hippeastrum latent virus in a big cluster where the majority of carlavirus CP sequences were grouped. At least five distinct minor clusters split from the main and more abundant group of species (Figure 3A). Their support by bootstrapping values was consistent, therefore, this represents the most updated phylogenetic description by the CP analysis in the *Carlavirus* genus. An alignment of some carlaviruses coat proteins, selected in principle by a closer phylogenetic affinity, was also prepared (Supplementary Materials Figure S3) to check for in silico evidence that could link JaVC to CPPMV in a putative similarity and therefore in a clue about vector transmission. The pairwise matrix returned percent values of 51.40, 52.10 and 52.78 similarity between CPPMV CP with those of JaVC-Bari, JavC A-31 and *Carya illinoiensis* carlavirus 1, respectively. An even higher value of identity (56.16% for JaVC A-31 and 56.85% for JaVC-Bari) was shared with Narcissus common latent virus CP. Instead, the identity between CPMMV and melon yellowing-associated virus coat proteins was 31.34%, and between both jasmine virus C isolates and shallot latent virus it was no more than 36%.

A very similar picture was observed about the replicase protein phylogenetic tree (Figure 3B), where the topology observed in the coat protein was essentially conserved. The outgroup most related protein was that from the foveavirus ASPV, while more distant were those from the tricho- and allexiviruses (ACLSV and GaVA, respectively). The conserved positions along the trees for all the subclusters was an indication that the evolutive differentiation in the carlavirus species was not perturbated by recombination events, at least for the genes considered in this analysis.

### 2.4. RT-PCR for JaVC Detection and Local Survey

To have a preliminary confirmation of the true presence of JaVC sequences in the three originally selected *J. officinale* sources, two sets of primers were designed in the first contigs obtained from nanopore sequencing of dsRNA extracts (R2; R3/5; Supplementary Materials Table S1). PCR products were amplified only from one clonal accession, while the other two candidate mother plants were negative. The conventional sequences from RT-PCR products clones were fully matching with the contig sequences (not shown). The positive amplification of the virus sequence in this accession was consistent with the presence of symptoms. Moreover, two more genomic fragments were amplified: one close to the 5’ end (a cloned fragment of 1300 bp) and the 3’ end co-terminal fragment of 243 bp (Supplementary Materials Table S1). Again, conventional sequences from these clones correctly aligned with assembled contigs (close to the 5’ end) and with the two matching JaVC Asian isolates (at the 3’ end noncoding region) (not shown).

Five further *J. officinale* accessions, sampled from different propagative batches in the same nursery, 12 months after the first selection of candidate mother plant accessions, were also tested by RT-PCR with the same primer sets (R2; R3/5). Three out of five plants (60%) positively reacted for JaVC-Bari presence showing both amplicons, while none of them had any evidence of symptoms.

## 3. Discussion

Several reads of the genomic sequence of a new isolate of JaVC were identified in a new host species (*J. officinale*) by nanopore sequencing, starting from a very little input amount of purified dsRNA. While the near full-length genome of the virus was then successfully assembled by Illumina sequencing, still to be considered a higher standard for the low error rate and more precise base calling, the potential of the nanopore high-throughput sequencing for the de novo virus discovery was confirmed in this study.

The de novo assembly of the reads originated from the nanopore sequencing of dsRNA (random-primed cDNA-PCR), and direct RNA do not work either using Canu or Flye [26,27]. These assemblers are calibrated to reconstruct complete microbial and near-complete eukaryotic genomes [26,27] and most likely are not suitable for the analysis of metagenomic libraries due to diversity and low coverage of retrieved reads as well as in terms of quality and quantity. Moreover, in our samples, viral reads represented a small amount of the total and were dispersed on the genomes, so the absence of consistent overlapping hampered the assembly step. Nevertheless, it cannot be excluded that a deeper sequencing and/or an enrichment of the viral fraction could overcome the limitations of these two assemblers in viral metagenomic studies.

Undoubtedly, the length and position of reads obtained from the direct RNA nanopore sequencing of a polyadenylated viral genome is a major gain of the technique. However, if the higher number of reads obviously starts from the polyA tail, we observed also several contigs scattered along the genome, and a minor priming role of small oligo-adenine stretches could not be ruled out for cDNA synthesis.

Reads obtained by the random-primed cDNA on the dsRNA are lesser in number and smaller in size. Either multiple priming sites, a fragmentation of genomic dsRNA molecules during the purification steps and the presence of sub-genomic RNA molecules acting as a template can account for this result.

Resuming the output of the two different nanopore sequencing approaches, the direct RNA system outreaches the random cDNA synthesis on identification of infectious viral agents in analyzed samples as length and number of reads. A negative effect of the direct RNA sequencing, which basically captures polyadenylated virus genomes, could be the loss of viral variability as non-polyadenylated viruses potentially escape the detection. The overall average quality of nanopore sequencing (Phred score 8, error rate ≥ 10%) could allow the identification at species level of discovered agents, but the error rate together with the very low coverage does not provide enough sequence material to perform intra-specific variability studies as well single-strain identification, recombination detection or other evolutionary analysis [28,29]. In this context, the need and support of another type of sequencing method (i.e., Sanger or Illumina) as a conclusive step of study on full-length genomes, along with the nanopore one, could be required [28,30,31].

Since we discovered the JaVC-Bari isolate in a different host species than that previously reported, genetic variability between this isolate and those sequenced in *J. sambac* was conceivably found. Indeed, with overall genomic structure and domain conservation, about 10 and 16% of amino acids were different in the CP and ORF1 polyprotein, respectively, among the Asiatic isolates and the Bari one. The highest similarity of around 75% (at amino acid level) with Hippeastrum latent virus in the CP, reported by Chang et al. [19], was confirmed by our data.

From the analysis of domains retrieved on JaVC gene sequences and the comparison of conserved motifs reported in the literature for carlavirus proteins, a few comments can be drawn. The replicase gene (ORF1) includes the AlkB region, associated with the 2-Oxoglutarate-Fe dioxygenase in a complex involved in RNA protection against methylation [32]. A similar ORF1 organization was recently ascertained by Jordan et al. on the full genome sequence of carnation latent virus [33]. The presence of the domain for the C23 endopeptidase implies the putative cleavage of the Tymo-like RdRp domain from the C-termination of a single polypeptide, with no evidence for a translational frameshift [34]. The conserved motif found in ORF2 is slightly different from the motif early reported as conserved in other carlaviruses homolog proteins (G-GKSS/T) [23]. A typical carlavirus signature C/TTTAGGT is located in the small intergenic regions upstream of both the triple gene block and the coat protein (positions 5973–5982 and 7177–7185, respectively) and it could have a suggested role as a ribosome-binding site for translation of subgenomic viral mRNAs [35].

It is worth noting the prediction in the CP of a structural N-term complex (an “intrinsic disorder” and a coil-domain, also found by Jordan et al., 2021 [33] analyzing the CLV CP by the same program), which could be an important feature for either protein–protein and protein–RNA interactions [24]. Up to date, a few studies faced the carlavirus CP sequence analysis [36,37], and this putative new function of the N-term complex, disclosed by innovative searching tools on updated databases [38], deserves a future investigation for its role in the virion assembly mechanism.

The ORF6-11kDa protein contains either a nuclear localization signal made up by a stretch of basic amino acids (R-X4-RKRR-X5-R) [39] and, immediately downstream of the latter motif, a cysteine-rich signature (C-X2-C-X12-C-X4-C) in the configuration of a C-4 “zinc finger” structure (Supplementary Materials Figure S1). Cysteine-rich proteins (CRP) in carlaviruses are known to act as nucleic acid-binding proteins (NABp) to regulate viral replication, behaving as a silencing suppressor, and are symptom determinants [40,41]. The presence of a nuclear localization signal in the NABp of a virus basically replicating in cytoplasm (like the carlavirus CVB studied by Lukovitskaya et al. [39]) has been associated with the upregulation of a transcription factor (*uppL*) linked to leaf malformation and cell proliferation. This is not the case, however, of JaVC infections so far described in jasmine, where most of the observed symptoms are yellow mosaic and discolorations. Role and function in replication activity of this domain in JaVC is again an open question.

Moreover, mild symptoms and latency could be considered as a consistent character of several carlaviruses in many environmental conditions [4] and could be linked to the weak silencing suppression activity of the CRPs [41]. Goshal and Sanfaçon [42] stated that viruses having a recovery mechanism (a significant symptom reduction or temporary latency) usually encode a weak silencing suppressor, so these viruses cannot face efficiently the silencing attack but can “hide” and persist below a low-level infection threshold needed for symptom expression. Indeed, symptoms observed on our accessions of *J. officinale* were not constantly evident in infected plants but apparently elicited by environmental conditions like those in greenhouse, while little or none of them were observed under open screens in nursery. Similar findings were reported about Coleus vein necrosis virus, which showed occasional yellow blotching, but never veinal necrosis, under average greenhouse conditions [43]. In our limited survey, run in a native nursery on some batches of *J. officinale*, only a part (60%) of the plants used in the nursery to start propagation was infected. Due to the quick turnover of propagative jasmine batches in the nursery, the conceivable care of nurserymen in eliminating any kind of symptomatic plants from further propagation and the periodical treatments against potential insect vectors, it could be understandable that not all the plant lots, chosen as starting propagative materials and maybe from a different origin, were certainly infected.

Confidently based on the high-throughput sequencing performance, no other viral agents were identified in our *J. officinale* sources, therefore, a strong association of observed symptoms to the presence of JaVC could be reasonably hypothesized. A carlavirus was already described to infect *J. sambac* in Italy in 1991 [17], associated with symptoms mainly in late summer, and a mixed infection with a potyvirid was also assessed by serological tests. Whether this carlavirus was the same agent found in our study in *J. officinale* is questionable. From a critical reading of [17], some evidence could be drawn: grafting infected *J. sambac* tissues on *J. officinale* did not give any positive transmission, and mechanical infection on herbaceous hosts was also unsuccessful. Finally, any kind of relationship with JCRSV [20] could not be discarded since we now know that some carlaviruses are transmitted through *Bemisia* spp. [44,45]. Since melon yellowing-associated virus and CPMMV are not serologically related despite the common vector [45], we searched for similarity with JaVC isolates and found that conserved stretches (between CPPMV and JaVC) accounted for slightly more than 50% identity. However, this hypothesis requires further investigation to fully assess vector transmission.

The presence of a potentially insect-transmissible virus is of concern among ornamental species anyway because of the large number of plants that could be latently infected and commercialized. The spread of the virus could find, by chance or by evolution, new vector species or new hosts, and only the capillary control on nursery productions or the establishment of virus-tested certified materials could avoid the escape from reservoirs of those potentially dangerous airborne agents.

## 4. Materials and Methods

### 4.1. Plant Material

**Figure 4:**
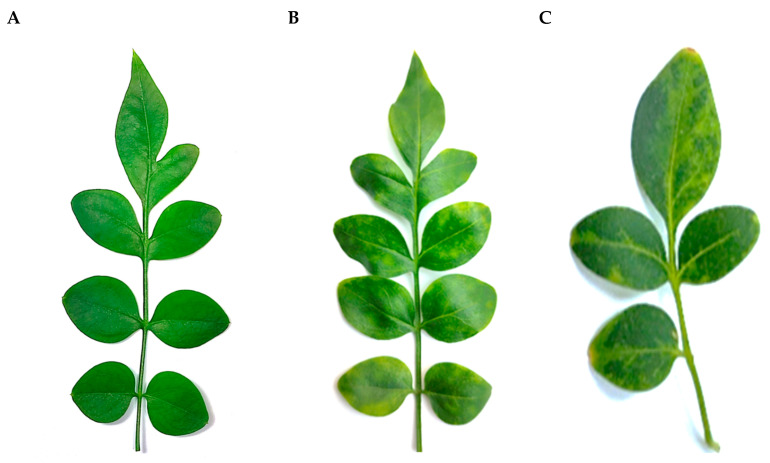
Leaves of *Jasminum officinale* showing symptoms of yellow spots and chlorotic variegation (**B**,**C**) and leaf of a healthy plant without symptoms (**A**).

Three *J. officinale* accessions, originated from vegetatively propagated batches and grown in a screen house of a commercial nursery (Monopoli, Apulia), were selected for general phytosanitary assessment in June 2020. When kept in our facilities (thermo-conditioned greenhouse at 25 °C and 80% humidity constant, 16 h light), one plant developed a mild interveinal mosaic and discoloration and sometimes yellow spots scattered on the leaf lamina (Figure 4). A pooled sample from the three sources was subjected to HTS analysis, along with other individually barcoded libraries from ornamental plants extracts, for detecting both known or not yet identified systemic pathogens without prior knowledge of specific target sequences.

### 4.2. Extraction of Nucleic Acid and Preparation of Libraries for MinION Sequencing

#### 4.2.1. Total RNA and dsRNA Extraction and Libraries Synthesis

Approximately 250 mg of leaf tissue was collected mixing the three *J. officinale* accessions, ground in liquid nitrogen and extracted in 1.5 mL guanidine isothiocyanate buffer, added with 4% Na-metabisulfite and 2% sarkosyl (NLS) [46].

Double-stranded RNAs were isolated to synthesize cDNA-PCR barcoded libraries, using two rounds of CC-41 cellulose (Whatman) microchromatography, washing with 18% ethanol in STE (sodium chloride, Tris, EDTA) 1x buffer. Partially purified dsRNA was resuspended at 25 ng/µL as quantified by Nanodrop (Implen NanoPhotometer^®^).

Before cDNA synthesis, extracted dsRNAs were denatured for 3 min at 98 °C, then snap-cooled on ice. First-strand cDNA was synthesized with Maxima H-Minus Reverse Transcriptase (Thermo Fisher Scientific) using a modified anchored random primer (5′-ACTTGCCTGTCGCTCTATCTTCNNNNNNNNN-3′) suitable for subsequent adapter annealing. The strand-switching method according to a SQK-PCB109 cDNA-PCR sequencing kit (Oxford Nanopore Technologies, ONT, Oxford, UK) was followed. cDNA synthesis and strand switching conditions were: 23 °C for 10 min; 50 °C for 10 min; 55 °C for 10 min; 42 °C for 10 min [47].

cDNAs were individually amplified by PCR with LongAmp Taq 2X Master Mix (New England Biolabs, Ipswich, USA) (with the barcoded primers in the ONT kit) and then cleaned with Agencourt AMPure XP beads (Beckman Coulter, Indianapolis, USA). Eluted libraries (1 µL) were observed in 1.2% agarose gel electrophoresis, and the total DNA amount was estimated using a Qubit DNA High Sensitivity Assay Kit (Thermo Fisher Scientific, USA) before sequencing.

Since we ran six differently barcoded libraries in the same experiment, they were equimolarly pooled, and 1 µL of adapter mix was added to the final pooled library. The mixture was then loaded onto the USB-powered MinION (Mk1B, MIN-101B) flow cell (Flo-Min 106d r9.4.1) for a run of 7 h.

#### 4.2.2. Direct RNA Sequencing

Total RNA from the same *J. officinale* tissues was extracted by the same procedure as above and then subjected to ribosomal RNA depletion (RiboMinus™ Plant Kit; Thermo Fisher Scientific, Waltham, MA, USA) to enrich the poly-adenilated fraction for cDNA synthesis.

A total of 500 ng of rRNA-depleted RNA, in a volume of 9 µL, was used for the library preparation following the direct RNA sequencing protocol for MinION (SQK-RNA002; ONT). Since many RNA viruses may have complex secondary structures, the RNA sample was denatured by heating for three minutes at 98 °C, then cooled down on ice for a few minutes.

Reverse transcription was made by adding SuperScript™ III reverse transcriptase (Thermo Fisher Scientific, USA) using an oligo-dT based primer. cDNA was cleaned up with RNAClean XP beads (Beckman Coulter, Indianapolis, IN, USA). One microliter of the adapter-ligated RNA-cDNA hybrid was quantified using a Qubit fluorometer DNA HS assay (Thermo Fisher Scientific, USA), before loading and sequencing the library in the flow cell as described above.

### 4.3. Total RNA Preparation for Illumina Sequencing

Total RNA was extracted from the same plant tissues using a Maxwell^®^ RSC Plant RNA Kit on Maxwell^®^ RSC Instruments (Promega, Madison, WI, USA) to minimize any possible contamination and enzyme inhibitors. DNA digestion was performed by adding 5 µL of DNAse I solution during the automatic purification steps. An aliquot of eluted RNA was visualized in 1.2% agarose gel electrophoresis. Total RNA was outsourced to the Institute of Applied Genomics (IGATech, Udine, Italy), where libraries were prepared using TruSeq Stranded Total RNA Sample Prep kits (Illumina, San Diego, CA, USA) after depletion of the rRNA fraction, and sequenced on an Illumina HiSeq 2000 platform in a 150 bp paired-end sequencing format.

### 4.4. Bioinformatics Analysis of Libraries

#### 4.4.1. Nanopore

The general quality of the nanopore libraries was estimated using PycoQC (https://hpc.nih.gov/apps/pycoQC.html; accessed on 5 June 2021 [48]). Total reads were quality-filtered using Epi2me software using the FASTQ CONTROL EXPERIMENT tool. Quality-checked reads were then further processed to remove sequences smaller than 20 nt using the fastx toolkit (http://hannonlab.cshl.edu/fastx_toolkit/; accessed on 5 June 2021). Processed reads were attempted to be de novo assembled with different parameters using either Canu or Flye [26,27] without obtaining a significant number of contigs. High-quality, longer reads were annotated using BLAST algorithm.

#### 4.4.2. Illumina

The quality of raw reads was assessed using the fastx toolkit (http://hannonlab.cshl.edu/fastx_toolkit/; accessed on 5 June 2021). Low-quality sequences (Phred ≤ 20, adapter, poly-N contamination and sequences smaller than 50 bp) were filtered out, and a trimming step of the 5′ ends was performed using cutadapt (https://cutadapt.readthedocs.io/en/stable/index.html; accessed on 5 June 2021 [49]). Reads passing such quality filters were then used for downstream analysis.

To unveil and characterize the virome of the sample under analysis, the sequenced total RNA dataset was de novo assembled using SPAdes v.3.15.3 [50]. Contigs were annotated using the BLAST algorithm [51].

#### 4.4.3. Annotation and Virome Characterization

Nanopore quality-filtered reads and Illumina de novo assembled contigs were both annotated using either BLASTn against the nt database (https://www.ncbi.nlm.nih.gov/nucleotide/; accessed on 5 June 2021) for the identification of known viruses or BLASTx against a customized database of viruses for the identification of possible new viral species. BLAST annotations were filtered based on significance e-value thresholds of 10^−6^ and 10^−4^ for BLASTn and BLASTx results, respectively.

To confirm the sequence in the 3’ terminal region (included the 3’ noncoding part), an oligo-dT-anchored primer was used to synthesize a 3’ terminal cDNA. A 3’ co-terminal fragment of 243 bp (excluding the polyA tract) was then amplified using the forward primer J3’for (Supplementary Materials Table S1) and the anchor-reverse primer. All the PCR products were cloned in a pSCa plasmid (StrataClone PCR Cloning Kit, Agilent, Santa Clara, CA, USA) and conventionally sequenced in both directions.

### 4.5. Intraspecific Variability and Phylogenesis

Since the only viral entity retrieved by the above analysis was the carlavirus jasmine virus C, paired-end reads from Illumina were aligned to an assembled JaVC genome through Bowtie2 v.2.3.5.1 using default parameters. The study of viral quasi-species was done by analyzing the number of single-nucleotide polymorphisms (SNPs) of reads mapping to the selected viral genomes. Coding regions in the retrieved viral genome were identified by ORFinder (NCBI), and all the genes were scanned for the content of annotated motifs and domains through InterPro [38].

Sequence comparison at nucleotide (among JaVC variants) and amino acidic (between JVC variants and other carlaviruses) level were performed through the Sequence Demarcation Tool (SDT v1.2; [52]) using the MUSCLE alignment algorithm [53]. Alignments of the aminoacidic residues of the JaVC ORF6-11kDa (available in GenBank) and coat proteins of some selected carlavirus species were done through the online Clustal Omega (https://www.ebi.ac.uk/Tools/msa/clustalo/; accessed on 5 June 2021).

The pairwise matrices, as well as phylogenetic relationships among our sequence and other carlaviruses, were evaluated using the replicase and coat protein amino acidic sequences. Sequences were first aligned using the MUSCLE algorithm, then, the best substitution model prediction was obtained, LG+G+I+F for the replicase sequences and LG+G for the coat proteins, respectively. Maximum likelihood phylogenetic trees were inferred in the MEGAX program with 1000 replicates of bootstrap.

### 4.6. Sequence Confirmation by RT-PCR

Two sets of primers were designed on preliminary carlavirus-related contigs obtained by MinION sequence (Supplementary Materials Table S1). Primers in contig R2, in the replicase gene, amplified a fragment of 164 bp, while primers designed on the alignment of partially overlapping contigs R3 and R5 (Supplementary Materials Table S1) produced an amplicon of 213 bp. A small preliminary survey to further investigate the presence of the virus in the same nursery was run 12 months later the first sampling on five *J. officinale* accessions (obtained by different propagative batches available in the nursery). Total RNA was reverse transcribed by random hexamers, and PCR-amplified [54] using the same sets of the above-described primers.

## Figures and Tables

**Table 1:** Nanopore sequencing (MinION, Oxford Nanopore) results of the barcoded cDNA-PCR library from dsRNA and direct RNA, before and after Epi2me quality controls.

	Raw Reads				Epi2me Quality Filtered Reads			
	Reads	Med Read Length	N50 Length	Med Read Quality	Reads	Med Read Length	N50 Length	Med Read Quality
cDNA-PCR from dsRNA	425,637	169	170	8.23	347,277	168	169	8.52
Direct RNA	328,990	652	1306	8.46	250,448	768	1326	8.89

## Data Availability

The full-length genome of Bari isolate of JaVC has been deposited in GenBank (Acc. nr. OL828237). All the sequencing output dataset generated in the study is freely available upon request to the Corresponding Author.

## Supplementary Materials

The following are available online at: https://www.mdpi.com/article/10.3390/plants11030309/s1, Supplementary Table S1: Specific primers designed on the genomic sequence of Jasmine virus C isolate Bari; Supplementary Table S2: List of all the viruses cited and used in the elaborations with available ICTV official abbreviations; Supplementary Table S3: Matrix of pairwise identities of coat protein amino acid sequences; Supplementary Table S4: Matrix of pairwise identities of replicase amino acid sequences; Supplementary Table S5: List of the most frequent nucleotide polymorphic sites retrieved in jasmine virus C-Bari consensus sequence; Supplementary Figure S1: Graphic representation of InterPro and CCD protein domain searches and annotations of jasmine virus C—Bari genome; Supplementary Figure S2: Alignment of the nucleic acid-binding proteins of JaVC available in GenBank; Supplementary Figure S3: Alignment of coat proteins of selected carlavirus members.
