# Supplementary material for: A New Jasmine Virus C Isolate Identified by Nanopore Sequencing Is Associated to Yellow Mosaic Symptoms of Jasminum officinale in Italy
Source: Plants (Basel). 2022 Jan 24;11(3):309. doi: 10.3390/plants11030309 (PMC8839810; doi:10.3390/plants11030309)
Supplement: Supplementary file 1 [file plants-11-00309-s001.zip › plants-1551306-supplementary.pdf]

A new jasmine virus C isolate identified by nanopore sequencing is associated to yellow mosaic symptoms of *Jasminum officinale* in Italy

Serafina Serena Amoia, Angelantonio Minafra, Vittorio Nicoloso, Giuliana Loconsole, Michela Chiumenti*

Institute for Sustainable Plant Protection – National Research Council, Via Amendola, 165/A, 70126 Bari - Italy

*Correspondending author: michela.chiumenti@ipsp.cnr.it (M.C.)

Supplementary Files

Supplementary Table S1.
Specific primers designed on the genomic sequence of jasmine virus C isolate Bari. The primers were used for RT-PCR initial confirmation on the infected plants, for viral detection in the survey and for sequencing of the 3' end portion (3' end RACE). Non-viral sequences are in italics. Nucleotide positions are on the consensus sequence deposited in GenBank (Acc. nr. OL828237).

Supplementary Table S2.
List of all the viruses cited and used in the elaborations (pairwise matrices, phylogenetic trees, Suppl. Figure S2) with available ICTV official abbreviations. The accession numbers as proteins ID (GenBank) or reference nucleotide genomes are reported.

Supplementary Table S3.
Matrix of pairwise identities of coat proteins amino acid sequences among jasmine virus C isolates and other member species in the *Carlavirus* genus.

Supplementary Table S4.
Matrix of pairwise identities of replicases amino acid sequences among jasmine virus C isolates and other member species in the *Carlavirus* genus.

Supplementary Table S5.
List of the most frequent nucleotide polymorphic sites retrieved in jasmine virus C Bari isolate genomic consensus sequence (produced by Illumina sequencing with an average coverage of 8.81x). Lower and higher frequency values are highlighted in yellow. Amino acidic residues changes are reported when a non-synonymous mutation is present in the codon (in red either the nucleotide change and amino acids of a different structural type).

Supplementary Figure S1.
Graphic representation of InterPro and CCD protein domain searches and annotations, including summary tables described in the genome of jasmine virus C Bari isolate. (A) ORF 1, Replicase domains. (B) ORF 2, Triple Gene Block 1. (C) ORF 3, Triple Gene Block 2. (D) ORF 4, Triple Gene Block 3. (E) ORF 5, Coat Protein. (F) ORF 6, Nucleic Acid Binding protein.

Supplementary Figure S2.
Alignment (obtained with Clustal Omega; https: //www.ebi.ac.uk/Tools/msa/clustalo/) of the nucleic acid binding proteins (ORF6 protein) of JaVC available in GenBank. Amino acid residues of the Bari isolate (from *J. officinale*) that diverge in the position from the almost invariant residues aligned from all the other isolates (from *J. sambac*) are in red. Accession numbers: Yunnan, MH389795; A31 Taiwan, KX364696; Fujian, MH231174; Guangdong, MH389788; Guangxi, MH389789; Hunan, MH389790; Jilin, MH389791; Sichuan, MH389793; Jangsu, MH389792; Shandong, MH389794.

Supplementary Figure S3.
Alignment (obtained with Clustal Omega; https ://www.ebi.ac.uk/Tools/msa/clustalo/) of coat proteins of selected *Carlavirus* members used to calculate the pairwise percentage similarity among aphid-transmitted and whitefly-transmitted species. Melon: melon yellowing-associated virus, MYaV, NC_038324; cowpea: cowpea mild mottle virus, CPPMV; carya: Carya illinoiensis carlavirus 1, MW328759; narcissus: Narcissus common latent virus, NCLV, NC_008266; A-31: jasmine virus C, Taiwan isolate A-31, KX364696; carnation: carnation latent virus, CLV, MN450069; shallot: shallot latent virus, SLV, NC_003557

Supplementary Table S1. Specific primers designed on the genomic sequence of jasmine virus C isolate Bari. The primers were used for RT-PCR initial confirmation on the infected plants, for viral detection in the survey and for sequencing of the 3' end portion (3' end RACE). Non-viral sequences are in italics. Nucleotide positions are on the consensus sequence deposited in GenBank (Acc. nr. OL828237).

**Primer Name**	**Sequence (5’ -> 3’)**	**Position (nt)**	**Product Size (bp)**
**Detection**			
Jvc_R2_for	5’-GAGCAAACTGCGGACTGATGCCT-3’	2975-2997	164
Jvc_R2_rev	5’-CTCTGATATTCTCTGCAGCCCTT-3’	3116-3138	
Jvc_R3/5_for	5’-CCTCAAACAGGATGCCGGCACTC-3’	7750-7772	213
Jvc_R3/5_rev	5’-GACTGATCAGGCGACCTACTCC-3’	7941-7962	
Jvc_rN3_for	5’-CCGCCGTTGTACATTCCCATCC-3’	268-289	1336
Jvc_r1_REV	5’-AGCACTTTCACCACCAATGCT-G	1582-1603	
**3’ RACE**			
J3'for	*5'*-GGGCGAAATCCATTTCTAGATG-*3'*	8249-8270	243
J3' cDNA RACE	5'-*GGCCACGCGTCGACTAGTACG*TTTTTTTTTTTTTTTTTTTTTN-3'		
J3-RACE Rev (Tag)	5'-*GGCCACGCGTCGACTAGTACG*-3'		

Supplementary Table S2. List of all the viruses cited and used in the elaborations (pairwise matrices, phylogenetic trees, Suppl. Figure S2) with available ICTV official abbreviations. The accession numbers as proteins ID (GenBank) or reference nucleotide genomes are reported.

Abbreviation	Species	Accession number CP	Accession number replicase
AcLV	Aconitum latent virus	BAB56118	NP_116487
ACLSV	Apple chlorotic leafspot virus	AAA42589	AAA42587
AHLV	American hop latent virus	AFI61523	YP_006297586
ASPV	Apple stem pitting virus	BAA04857	BAA04853
AtrMoV	Atractylodes mottle virus	AKZ66618	YP_009508317
BlSV	Blueberry scorch virus	NP612812	AAY18403
ButMV	Butterbur mosaic virus	BAI49696	QQX32740
CLV	Carnation latent virus	CAA09306	QJX15400
-	Carya illinoinensis carlavirus 1	QQG34676	QQG34672
CVB	Chrysanthemum virus B	CAM35757	CAO78688
CVR	Chrysanthemum virus R	YP009553180	YP_009553176
CoLV	Cole latent virus	QGN03517	QGN03513
CoMMV	Cole mild mosaic virus	QGN03509	QGN03507
CVNV	Coleus vein necrosis virus	ABS89249	EF527260
CPMMV	Cowpea mild mottle virus	ADQ54111	HQ184471
DVS	Daphne virus S	CAF04331	BAU20378
ElVC	Elderberry carlavirus C	YP009224944	YP_009224940
ElVE	Elderberry carlavirus E	ALY33533	YP_009224952
GalLV	Gaillardia latent virus	AHN84515	YP_009022064
GaVA	Garlic virus A	BAA61810	BAA61806
HelVS	Helenium virus S	BAA01248	QQX32728
HNNV	Helleborus net necrosis virus	ACM45983	FJ196835
HLV	Hippeastrum latent virus	AAZ15110	YP_002308447
HpLV	Hop latent virus	BAB13716	AJR19303
HpMV	Hop mosaic virus	ACB32253	YP_001798592
HdCMV	Hydrangea chlorotic mottle virus	ACE78185	YP_002985636
KLV	Kalanchoe latent virus	ACL01038	FJ531634
LNRSV	Ligustrum necrotic ringspot virus	ABW69738	QUP51755
LVA	Ligustrum virus A	AOO96603	YP_009288956
LSV	Lily symptomless virus	CAD56794	QBS17038
MYaV	Melon yellowing-associated virus	NC_038324.1	-
MjMV	Mirabilis jalapa mottle virus	AEQ35304	YP_004901680
NCLV	Narcissus common latent virus	CAC85384	YP_699983
NCLV	Narcissus common latent virus	YP_699987	CAJ43609
OVH	Opuntia virus H	ANH10858	ANH10854
PLV	Passiflora latent virus	ABE97939	QWT83583
PhlVB	Phlox Virus B	ABW05096	YP_001552317
PhlVM	Phlox virus M	YP_009664747	
PhlVS	Phlox virus S	ABP37860	YP_001165301
PVM	Potato virus M	BAA03343	QBL75476
PVM	Potato virus M	ASV70527	-
PVS	Potato virus S	CAI06119	QNQ79442
SLV	Shallot latent virus	NC_003557.1	-
-	Stevia carlavirus 1	QQG34563	QQG34559
-	Tagetes carlavirus 1	QQG34557	QQG34553
YLV	Yam latent virus	AJD23369	YP_009134730

Supplementary Table S3.

Matrix of pairwise identities of coat proteins amino acid sequences among jasmine virus C isolates and other member species in the *Carlavirus* genus.

>American_hop_latent_virus_AFI61523	100.001	2	3	4	5	6	7	8	9	10	11	12	13	14	15	16	17	18	19	20	21	22	23	24	25	26	27	28	29	30	31	32	33	34	35	36	37B	38T	39F	40	41	42	43	44	45	46	47
>Elderberry_carlavirus_E_ALY33533	65.401	100.001																																													
>Elderberry_carlavirus_C_YP009224944	67.401	81.301	100.001																																											
>Hydrangea_chlorotic_mottle_virus_ACE78185	44.101	46.101	45.601	100.001																																									
>Ligustrum_necrotic_ringspot_virus_ABW69738	43.901	46.401	48.001	58.901	100.001																																								
>Mirabilis_jalapa_mottle_virus_AEQ35304	42.701	46.901	46.001	67.201	64.001	100.001																																							
>Kalanchoe_latent_virus_ACL01038	46.601	48.401	47.301	68.401	66.901	79.901	100.001																																						
>Lily_symptomless_virus_CAD56794	44.301	47.901	48.101	65.901	68.101	77.301	76.301	100.001																																					
>Passiflora_latent_virus_ABE97939	48.101	50.301	50.701	65.701	70.601	77.301	75.001	73.801	100.001																																				
>Blueberry_scorch_virus_NP612812	44.201	46.401	47.301	68.501	68.101	69.901	72.501	72.201	74.501	100.001																																			
>Opuntia_virus_H_ANH10858	43.101	47.101	45.801	67.101	69.701	72.301	76.401	72.401	73.901	74.001	100.001																																				
>Potato_virus_S_CAI06119	47.501	45.701	45.601	62.401	65.801	71.101	70.801	66.301	68.901	69.001	67.001	100.001																																			
>Ligustrum_virus_A_AOO96603	41.501	43.201	44.601	45.801	48.001	49.101	47.501	46.001	47.701	46.601	48.301	44.501	100.001																																		
>Stevia_carlavirus_1_QQG34563	41.701	43.001	41.201	44.101	47.001	43.001	42.201	44.101	43.701	45.901	43.901	45.101	59.501	100.001																																	
>Tagetes_carlavirus_1_QQG34557	39.401	42.401	42.801	44.501	45.401	44.301	43.301	42.601	45.101	46.301	48.101	44.701	61.201	75.901	100.001																																
>Helenium_virus_S_BAA01248	38.701	41.601	41.601	43.101	46.101	44.801	44.701	43.601	45.501	45.801	47.501	46.601	66.201	66.801	67.701	100.001																															
>Atractylodes_mottle_virus_AKZ66618	38.301	40.501	41.601	43.201	46.501	41.801	43.701	44.001	45.501	42.401	47.601	42.201	58.501	61.301	58.601	57.401	100.001																														
>Chrysanthemum_virus_B_CAM35757	39.601	43.001	44.201	45.401	48.501	45.001	47.201	45.001	48.601	45.001	47.301	46.101	60.801	57.801	61.501	62.001	65.501	100.001																													
>Phlox_Virus_B_ABW05096	37.201	41.201	40.901	43.201	46.501	41.501	45.001	43.001	44.101	42.201	46.101	44.801	61.101	58.201	60.201	54.801	57.201	60.301	100.001																												
>Gaillardia_latent_virus_AHN84515	36.301	41.101	39.701	46.601	44.701	43.901	46.101	47.201	45.801	43.301	46.001	44.901	61.101	56.401	60.301	59.501	52.101	53.201	55.801	100.001																											
>Phlox_virus_S_ABP37860	35.901	45.601	41.301	45.201	42.801	48.101	47.501	44.301	46.201	43.401	45.701	48.101	58.301	53.401	59.901	58.601	56.301	58.201	54.901	75.501	100.001																										
>Daphne_virus_S_CAF04331	39.101	43.001	45.501	45.301	48.801	43.001	47.201	47.401	48.301	45.501	47.301	48.801	62.901	60.101	60.701	59.201	58.801	55.201	57.101	54.001	57.901	100.001																									
>Carya_illinoinensis_carlavirus_1_QQG34676	40.701	44.301	44.801	46.901	48.701	45.401	47.701	47.201	49.501	45.801	45.501	45.101	47.401	49.101	48.801	49.701	46.701	50.201	45.701	51.601	49.701	50.001	100.001																								
>Chrysanthemum_virus_R_YP009553180	39.901	46.301	45.801	46.501	48.801	45.201	48.101	46.601	48.801	44.801	45.901	44.701	47.101	47.101	46.201	47.001	44.301	47.201	43.301	48.401	48.601	48.201	88.301	100.001																							
>Cowpea_mild_mottle_virus_ADQ54111	41.101	45.001	43.701	47.201	46.701	48.101	47.701	45.601	47.501	46.001	46.301	44.501	46.701	46.601	45.501	44.601	46.001	43.701	47.701	47.901	51.001	46.301	51.401	54.201	100.001																						
>Hop_mosaic_virus_ACB32253	41.201	47.301	45.101	46.901	50.501	47.701	50.501	46.901	49.801	48.001	47.801	50.001	55.201	49.701	49.701	50.501	44.101	45.501	49.701	46.601	49.501	50.501	50.801	51.301	51.401	100.001																					
>Yam_latent_virus_AJD23369	42.501	47.001	46.101	44.601	48.201	44.301	48.601	47.401	48.601	46.101	45.701	48.601	52.401	49.301	49.101	51.901	45.701	46.001	46.801	46.101	48.301	51.101	52.601	51.101	50.701	64.501	100.001																				
>AM158439_Narcissus_common_latent_virus_QDVZC7	42.601	45.401	45.801	47.301	49.501	48.001	48.901	48.101	48.301	49.301	48.801	48.601	49.701	50.001	48.501	47.801	47.001	44.701	47.801	45.601	45.901	51.201	54.701	52.701	52.301	63.301	67.401	100.001																			
>Narcissus_common_latent_virus_CAC85384	42.701	48.001	46.801	47.601	51.001	49.301	49.301	50.901	50.001	48.301	47.301	48.001	50.301	48.601	50.701	48.801	45.701	46.001	48.301	46.401	48.301	50.301	53.001	53.301	49.701	65.001	68.401	87.401	100.001																		
>|Potato_virus_M_BAA03343	40.301	47.601	45.301	44.401	45.801	44.901	44.901	47.101	46.201	44.601	45.001	46.201	49.701	49.301	47.401	49.801	46.001	46.801	45.301	46.301	46.101	47.501	51.201	49.801	53.001	64.601	66.401	69.801	71.101	100.001																	
>Potato_virus_M_ASV70527	42.201	47.401	46.301	44.201	47.801	48.001	46.101	48.101	48.301	46.701	46.501	46.901	51.401	49.801	49.301	50.201	46.801	44.601	46.801	47.301	49.101	52.201	52.301	50.301	53.001	63.001	66.701	69.801	71.101	84.901	100.001																
>Cole_mild_mosaic_virus_QGN03509	41.801	44.701	45.801	45.401	51.201	46.701	48.901	48.501	49.101	47.201	47.301	46.901	49.701	48.001	49.101	48.301	42.801	42.801	47.301	44.701	45.601	50.001	56.801	55.701	53.101	67.701	66.201	73.201	72.401	75.701	74.301	100.001															
>Cole_latent_virus_QGN03517	41.201	47.101	46.901	46.201	48.801	47.701	48.601	48.101	49.301	46.501	47.301	47.601	49.001	48.601	49.101	48.801	43.801	46.501	47.301	44.701	45.901	48.701	56.601	55.301	51.701	66.101	66.901	72.801	72.101	75.001	72.401	81.001	100.001														
>Phlox_virus_M_YP_009664747	42.601	44.201	46.101	47.201	49.501	48.801	50.001	48.501	49.801	47.801	49.501	49.001	52.101	50.201	48.101	51.501	44.801	47.501	48.101	45.301	45.101	51.301	54.201	53.201	53.101	64.501	67.201	71.401	70.401	71.501	72.201	74.201	73.601	100.001													
>Aconitum_latent_virus_BAB56118	41.401	45.301	46.101	46.301	49.501	48.501	50.901	49.501	50.301	49.501	49.501	49.101	52.101	52.901	49.801	51.001	45.801	48.501	50.201	46.201	47.301	50.301	53.501	56.101	52.401	66.601	69.101	71.801	71.601	69.401	70.401	72.001	71.301	72.401	100.001												
>Hop_latent_virus_BAB13716	42.901	45.501	47.301	44.601	49.301	46.901	49.101	47.601	51.901	46.901	47.101	47.401	49.801	49.501	49.701	49.001	44.001	44.901	48.301	43.901	47.101	52.001	51.601	52.301	51.901	64.901	64.401	65.001	65.001	63.401	63.401	69.701	66.901	67.801	68.001	100.001											
>Jasmine_virus_C_J_officinalis_BA	42.701	45.001	45.801	47.301	51.201	49.501	50.201	49.101	50.501	50.701	50.901	47.401	49.101	46.401	46.401	48.801	42.801	44.101	47.801	47.501	49.301	46.101	49.301	48.501	54.501	56.801	55.701	56.801	58.601	58.301	60.701	58.601	57.401	58.101	58.301	56.801	100.001										
>Jasmine_virus_C_isolate_A31_AON75924	42.501	46.001	46.201	44.501	51.201	48.101	47.301	48.401	50.201	49.001	49.801	47.801	50.501	46.401	46.001	49.101	42.401	43.601	46.801	47.501	46.101	46.801	49.001	48.301	53.301	55.401	55.701	55.101	59.201	59.001	61.001	56.401	56.401	56.101	57.301	54.401	90.901	100.001									
>Jasmine_virus_C_isolate_Fujian_QAA12646	43.201	45.801	46.501	48.501	51.401	48.801	48.801	48.801	50.501	50.201	52.301	48.101	49.701	46.401	45.901	47.401	41.701	43.301	47.101	47.101	47.101	48.101	49.301	49.301	52.101	56.801	55.401	56.301	57.801	59.701	61.401	58.601	56.401	56.401	58.401	55.601	90.901	94.601	100.001								
>Hippeastrum_latent_virus_AAZ15110	45.001	48.601	46.901	46.901	52.201	50.201	50.201	47.901	49.801	49.101	49.501	48.501	51.401	49.001	47.901	51.401	43.601	46.501	45.001	48.101	51.001	48.101	52.801	51.001	54.001	59.801	61.501	59.701	61.601	61.301	64.101	60.301	60.301	59.901	59.201	59.601	75.701	74.301	75.301	100.001							
>Carnation_latent_virus_CAA09306	34.001	36.201	38.801	38.701	42.801	39.801	42.401	38.801	42.301	41.201	41.001	39.601	42.801	39.201	41.201	38.701	38.201	37.201	39.401	38.601	42.001	39.301	38.201	38.901	43.301	41.601	36.401	37.701	41.401	39.801	41.001	37.301	39.301	42.901	38.101	40.301	40.301	40.901	40.701	39.901	100.001						
>Butterbur_mosaic_virus_BAI49696	36.701	38.301	38.301	38.301	35.701	37.701	39.101	36.601	41.301	36.301	39.301	36.901	39.201	38.001	39.501	37.901	35.401	35.301	37.701	38.501	40.101	37.001	38.901	38.701	42.101	39.201	38.601	41.401	40.501	39.101	41.601	40.101	39.801	40.001	41.901	40.101	39.701	38.001	38.601	40.901	65.001	100.001					
>Coleus_vein_necrosis_virus_ABS89249	33.801	34.501	34.801	38.201	36.301	35.401	38.701	36.801	39.201	39.101	39.101	38.101	38.901	39.701	39.101	37.601	36.301	34.801	39.101	37.801	38.001	38.901	37.701	38.401	39.401	37.201	35.801	40.901	41.801	37.901	38.401	39.501	39.101	39.901	38.101	36.101	36.901	38.301	38.301	42.601	62.701	56.901	100.001				
>Helleborus_net_necrosis_virus_ACM45983	34.901	37.201	38.401	41.501	39.801	42.201	42.601	39.201	41.801	41.601	44.801	38.901	41.101	37.401	39.601	37.401	34.001	34.601	37.201	39.401	39.901	39.301	40.201	42.101	38.001	42.101	39.801	40.301	40.701	37.701	40.001	40.101	42.001	42.501	37.701	40.701	41.701	40.301	40.801	41.901	49.701	47.601	46.901	100.001			
>Apple_stem_pitting_virus_BAA04857	28.401	28.701	30.601	31.601	30.801	26.601	27.701	29.201	32.201	28.301	30.801	29.501	27.301	29.601	31.901	27.801	31.101	28.901	28.501	30.001	32.101	32.601	29.501	29.101	33.001	30.201	32.001	29.701	33.601	31.201	32.201	33.701	29.501	29.801	33.301	27.601	29.501	28.901	29.701	32.501	32.101	30.401	29.501	29.101	100.001		
>Garlic_virus_A_BAA61810	22.001	25.201	23.801	21.901	21.301	23.301	19.901	19.501	20.501	21.601	22.401	26.101	21.501	24.601	20.601	24.901	25.101	23.801	23.201	22.001	26.901	22.601	19.401	19.001	21.201	24.401	25.701	24.401	24.001	24.801	22.401	24.501	25.301	23.901	20.501	20.301	24.501	24.901	27.201	23.701	21.601	27.501	27.601	25.001	29.401	100.001	
>Apple_chlorotic_leaf_spot_virus_AAA42589	22.601	23.801	22.501	20.701	17.601	21.701	19.501	16.601	17.201	17.701	21.601	19.201	22.801	22.301	20.901	19.801	20.901	20.401	20.901	22.501	21.501	25.401	21.901	21.101	24.501	20.301	19.301	16.501	21.001	24.201	26.101	21.501	22.101	24.601	21.401	21.201	21.901	22.001	20.901	21.101	22.201	24.001	19.601	17.301	25.601	15.301	100.001

Supplementary Table S4.

Matrix of pairwise identities of replicases amino acid sequences among jasmine virus C isolates and other member species in the *Carlavirus* genus.

>Helleborus_net_necrosis_virus_ACM45979__	100.001																																											
>Carya_illinoinensis_carlavirus_1_QQG34672	47.701	100.001																																										
>Chrysanthemum_virus_R_YP009553176	48.601	78.301	100.001																																									
>Chrysanthemum_virus_B_CAO78688	46.701	63.201	68.501	100.001																																								
>Ligustrum_virus_A_YP009288956	47.801	49.801	50.101	51.601	100.001																																							
>Daphne_virus_S_BAU20378	47.401	48.501	49.301	50.501	54.501	100.001																																						
>Phlox_virus_B_YP_001552317	47.401	48.601	48.701	50.701	52.601	49.701	100.001																																					
>Gaillardia_latent_virus_YP009022064	46.601	49.101	49.601	53.001	53.701	50.301	56.101	100.001																																				
>Phlox_virus_S_YP001165301	47.401	48.701	48.901	52.701	53.101	50.801	56.701	70.701	100.001																																			
>Stevia_carlavirus_1_QQG34559	47.101	50.301	50.101	53.801	51.801	51.701	56.601	55.701	56.601	100.001																																		
>Tagetes_carlavirus_1_QQG34553	47.301	49.101	49.801	53.201	53.201	49.501	56.001	56.401	57.401	69.201	100.001																																	
>Helenium_virus_S_QQX32728	47.701	49.001	49.501	52.301	51.901	50.301	55.401	57.201	56.101	58.901	58.101	100.001																																
>Atractylodes_mottle_virus_YP009508317	46.101	49.501	49.001	53.401	51.701	49.301	53.401	54.301	53.701	56.901	55.701	55.801	100.001																															
>Jasmine_virus_C_J_officinalis_BA	47.701	49.701	49.101	49.401	50.301	49.001	49.701	49.101	48.201	49.101	49.101	48.901	48.301	100.001																														
>Jasmine_virus_C_isolate_A31_AON75924	47.401	48.701	49.601	49.701	50.701	48.501	49.301	49.601	48.601	49.201	49.001	49.701	47.901	84.301	100.001																													
>Jasmine_virus_C_isolate_Fujian_QAA12646	46.701	49.001	50.401	49.401	50.201	48.701	48.901	48.801	48.701	49.001	48.901	49.201	48.301	84.001	92.101	100.001																												
>Hippeastrum_latent_virus_YP002308447	44.801	48.001	47.501	47.601	47.901	47.001	48.101	47.401	48.801	48.301	47.801	47.401	47.801	57.401	58.501	58.001	100.001																											
>Yam_latent_virus_YP009134730	45.801	48.201	49.301	48.301	50.101	48.001	49.101	49.601	48.901	48.801	47.901	49.401	48.201	51.601	51.501	50.701	48.501	100.001																										
>Hop_mosaic_virus_YP001798592	46.401	48.801	48.401	48.401	51.201	47.301	50.101	48.801	49.601	50.401	49.501	49.001	50.601	51.401	51.201	51.701	50.301	63.101	100.001																									
>Hop_latent_virus_AJR19303	45.101	48.701	49.701	48.401	49.701	47.101	48.701	49.401	49.201	49.101	49.201	47.801	48.301	49.801	50.301	50.501	48.601	61.201	61.701	100.001																								
>Narcissus_common_latent_virus_YP699983	46.101	48.801	49.001	48.201	48.801	47.801	47.901	48.901	48.001	49.101	48.401	48.301	49.001	49.701	50.001	49.601	48.901	57.201	57.301	57.301	100.001																							
>Potato_virus_M_BAA03339	43.701	45.601	45.401	45.901	47.001	44.501	45.601	45.301	46.601	46.301	46.401	46.701	46.301	47.501	48.301	47.101	49.101	55.001	54.901	54.501	56.301	100.001																						
>Cole_mild_mosaic_virus_QGN03507	44.501	46.701	47.001	46.901	48.401	46.201	47.501	46.801	47.701	48.301	47.801	46.101	46.501	48.701	49.501	50.001	49.901	57.201	57.701	56.601	60.001	59.801	100.001																					
>Cole_latent_virus_QGN03513	46.201	46.601	46.201	46.201	48.401	46.201	46.201	46.701	46.801	47.501	46.901	47.001	47.801	48.301	48.501	48.601	47.901	57.201	57.301	56.401	59.801	59.501	71.001	100.001																				
>Aconitum_latent_virus_NP116487	44.901	47.601	48.101	47.301	49.201	46.401	47.501	48.101	47.301	48.801	48.801	48.801	47.401	50.301	50.001	49.601	49.201	57.001	58.601	56.001	55.801	53.201	54.801	55.101	100.001																			
>Cowpea_mild_mottle_virus_ADQ54107	45.901	46.801	47.901	48.601	49.701	45.901	49.201	48.601	48.301	49.201	48.601	48.301	48.801	49.001	49.501	48.501	50.201	49.901	51.301	49.301	49.201	48.801	48.601	48.401	49.701	100.001																		
>American_hop_latent_virus_YP006297586	48.701	47.801	48.901	49.201	50.501	49.901	51.001	50.401	50.201	51.101	50.201	50.001	49.501	48.601	49.201	47.901	47.301	48.801	47.801	48.301	49.101	46.101	46.901	46.801	48.501	49.401	100.001																	
>Elderberry_carlavirus_E_YP009224952	48.501	49.001	48.901	48.701	50.601	49.001	49.701	48.501	49.901	49.101	49.901	50.601	50.501	48.401	48.501	48.001	46.801	47.501	48.001	47.801	46.501	44.501	45.301	46.001	47.101	49.101	59.901	100.001																
>Elderberry_carlavirus_C_YP009224940	48.601	47.501	48.901	48.301	51.201	49.101	48.901	50.201	50.101	51.801	50.601	50.301	49.401	48.401	49.101	48.701	46.001	47.701	48.101	47.801	47.201	44.701	46.501	46.501	48.901	47.401	58.601	61.801	100.001															
>Hydrangea_chlorotic_mottle_virus_YP002985636	47.601	48.901	48.501	48.201	49.301	48.401	49.701	49.701	49.101	48.601	48.601	49.201	48.601	48.501	48.701	48.901	47.501	49.001	49.001	47.701	48.001	45.901	46.301	44.901	47.801	48.701	52.101	49.701	51.001	100.001														
>Blueberry_scorch_virus_AAY18403	46.901	49.001	47.701	48.101	49.001	47.901	48.201	49.201	48.801	48.901	48.801	48.201	48.401	48.101	48.301	48.001	46.701	47.701	48.301	47.901	46.501	44.801	47.401	46.001	46.901	49.301	50.801	50.201	50.701	59.001	100.001													
>Opuntia_virus_H_ANH10854	46.601	47.501	47.301	46.901	49.001	47.401	48.001	48.901	48.601	48.401	48.101	48.301	48.401	48.201	48.601	47.901	45.601	47.601	47.801	47.601	46.701	45.301	45.001	45.001	47.401	47.801	50.301	49.301	49.601	58.401	58.301	100.001												
>Ligustrum_necrotic_ringspot_virus_QUP51755	47.601	47.501	46.901	46.901	48.601	46.701	47.501	48.501	47.901	48.001	48.101	48.201	48.401	48.401	48.501	48.301	45.901	47.101	47.101	46.101	46.301	44.601	45.401	44.401	46.401	47.701	50.301	48.801	48.901	57.001	57.701	56.501	100.001											
>Mirabilis_jalapa_mottle_virus_YP004901680	45.701	48.001	47.701	47.001	47.401	47.901	48.701	49.001	48.201	48.701	48.501	49.001	48.501	48.501	47.901	48.301	46.101	48.201	49.201	48.401	46.601	44.701	45.601	45.101	46.901	48.301	49.201	49.601	49.301	57.301	57.601	57.701	57.601	100.001										
>Passiflora_latent_virus_QWT83583	45.201	47.201	47.301	46.701	47.801	47.301	47.301	48.201	46.901	47.601	48.301	47.601	48.201	48.101	48.001	47.901	46.301	48.201	46.701	48.001	47.301	45.701	45.501	45.401	46.301	47.501	49.701	49.001	49.101	57.401	56.801	57.601	56.501	60.301	100.001									
>Kalanchoe_latent_virus_ACL01033	46.401	46.501	46.501	45.601	48.601	47.101	47.401	48.201	47.801	48.301	48.401	47.801	48.301	48.901	48.801	48.601	45.901	45.901	47.001	47.101	45.801	43.201	44.901	44.101	46.301	47.401	50.401	49.401	49.901	59.101	55.501	57.901	57.201	60.501	60.401	100.001								
>Lily_symptomless_virus_QBS17038	45.601	47.701	47.801	47.001	47.501	46.401	47.801	49.001	48.201	48.301	48.801	47.801	47.401	47.301	48.001	47.801	47.101	46.901	48.001	46.801	45.101	43.501	44.901	44.101	47.201	47.701	50.101	49.701	49.001	58.601	56.601	56.601	57.501	60.101	59.401	59.801	100.001							
>Potato_virus_S_QNQ79442	46.001	45.301	46.001	45.701	47.001	46.301	45.501	47.301	47.101	47.201	47.001	46.301	46.801	46.501	46.901	46.901	44.801	47.601	46.601	45.001	45.801	44.101	45.501	45.001	44.901	45.901	48.701	48.301	47.901	53.901	53.001	52.401	53.801	52.701	52.401	52.401	51.101	100.001						
>Carnation_latent_virus_QJX15400	45.501	44.301	45.301	44.701	46.501	44.301	43.501	45.401	44.001	44.801	44.601	44.601	45.401	43.901	44.501	44.401	42.601	43.401	43.601	43.301	43.401	42.801	42.901	44.001	43.901	44.501	45.601	43.301	44.101	44.701	44.701	44.401	46.001	43.401	43.501	44.501	43.601	43.501	100.001					
>Butterbur_mosaic_virus_QQX32740	43.901	43.601	43.301	43.401	46.801	45.201	44.501	44.601	44.001	44.901	45.301	44.301	44.401	45.601	44.801	45.001	42.001	44.601	44.401	43.901	43.301	41.301	43.101	42.901	43.701	43.901	44.101	44.401	45.301	45.201	45.101	45.901	46.001	44.101	43.501	44.501	44.601	44.401	47.501	100.001				
>Coleus_vein_necrosis_virus_ABS89245	46.101	44.801	44.101	43.501	46.601	44.301	44.101	45.201	44.601	45.301	46.101	46.501	44.801	43.901	43.701	43.901	42.501	44.701	45.301	44.101	44.701	42.101	43.201	43.401	43.601	45.201	44.901	44.101	45.001	44.701	44.601	43.801	44.701	44.701	43.201	44.901	43.301	43.201	47.001	48.001	100.001			
>Apple_stem_pitting_virus_BAA04853	43.701	42.001	41.001	40.901	44.401	41.701	42.401	43.401	42.501	43.801	43.901	43.501	44.001	43.201	43.501	43.101	41.701	43.401	42.001	42.701	43.101	41.901	43.301	40.701	43.201	43.801	42.301	43.901	43.801	42.901	44.101	42.301	43.501	43.801	42.801	43.001	42.201	41.101	41.401	41.601	41.301	100.001		
>Apple_chlorotic_leaf_spot_virus_AAA42587	34.101	32.401	32.201	31.701	33.001	32.801	33.701	34.601	33.101	32.701	33.201	33.201	33.701	34.201	34.401	33.501	32.101	35.001	33.401	32.401	34.901	32.201	32.501	32.201	32.301	32.701	34.201	33.501	33.601	32.301	32.601	32.601	33.301	32.001	32.601	32.301	33.001	31.101	32.401	31.401	32.801	33.501	100.001	
>Garlic_virus_A_BAA61806	24.101	24.401	26.701	25.901	25.501	25.301	24.101	25.101	25.001	26.201	25.301	25.701	26.301	26.001	25.601	25.601	26.101	26.101	25.901	24.501	25.901	24.701	25.601	25.301	26.701	25.401	25.401	25.701	26.101	25.501	25.001	25.901	25.201	24.201	25.801	24.901	25.501	26.201	23.701	25.501	24.901	25.901	24.301	100.001

Supplementary Table S5.

List of the most frequent nucleotide polymorphic sites retrieved in jasmine virus C Bari isolate genomic consensus sequence (produced by Illumina sequencing with an average coverage of 8.81x). Lower and higher frequency values are highlighted in yellow. Amino acidic residues changes are reported when a non-synonymous mutation is present in the codon (in red either the nucleotide change and amino acids of a different structural type).

Name	Gene	Position	Length	Change	Polymorphism Type	Variant Frequency	P-Value	Codon	Amino acid	Changes	
T	ORF1	668	1	C -> T	SNP (transition)	19.7%	2.7E-33	GAc	D=D		
G	"	716	1	A -> G	SNP (transition)	11.9%	1.8E-26	GTa	V=V		
T	"	941	1	C -> T	SNP (transition)	16.7%	1.6E-63	GTc	V=V		
C	"	1001	1	T -> C	SNP (transition)	11.7%	3.4E-19	CCt	P=P		
T	"	1052	1	C -> T	SNP (transition)	48.6%	2.3E-128	AGc	S=S		
T	"	1250	1	C -> T	SNP (transition)	23.2%	2.1E-73	GGc	G=G		
G	"	1525	1	C -> G	SNP (transversion)	19.8%	2.3E-50	AcC	T	AgC	S
T	"	1544	1	C -> T	SNP (transition)	11.0%	7.5E-25	TTc	F=F		
A	"	1617	1	T -> A	SNP (transversion)	13.8%	3.0E-29	tGC	C	aGC	S
G	"	1908	1	A -> G	SNP (transition)	25.4%	1.2E-36	aAA	K	gAA	E
G	"	1940	1	A -> G	SNP (transition)	10.5%	1.4E-13	AGa	R=R		
A	"	2046	1	G -> A	SNP (transition)	10.8%	1.4E-27	TTg	L=L		
T	"	2432	1	C -> T	SNP (transition)	17.6%	2.8E-42	GTc	V=V		
A	"	3086	1	T -> A	SNP (transversion)	12.4%	1.6E-31	CGt	R=R		
A	"	3242	1	T -> A	SNP (transversion)	11.3%	8.7E-22	GTt	V=V		
A	"	3278	1	G -> A	SNP (transition)	10.0%	2.1E-12	GAg	E=E		
G	"	3485	1	A -> G	SNP (transition)	49.5%	3.9E-139	CTa	L=L		
T	"	3560	1	C -> T	SNP (transition)	16.8%	3.3E-34	CAc	H=H		
G	"	3944	1	A -> G	SNP (transition)	54.5%	2.1E-150	ACa	T=T		
G	"	4004	1	A -> G	SNP (transition)	16.4%	4.5E-27	CGa	R=R		
G	"	4245	1	**A -> G**	SNP (transition)	10.1%	2.8E-20	aTC	I	gTC	V
C	"	4417	1	T -> C	SNP (transition)	17.3%	1.4E-40	GtG	V	GcG	A
C	"	4538	1	T -> C	SNP (transition)	45.5%	5.4E-81	GGt	G=G		
A	"	4844	1	C -> A	SNP (transversion)	13.9%	1.1E-19	GTc	V=V		
C	"	5084	1	T -> C	SNP (transition)	14.2%	6.1E-31	GGt	G=G		
C	"	5165	1	T -> C	SNP (transition)	36.7%	1.0E-106	TGt	C=C		
C	"	5729	1	T -> C	SNP (transition)	11.9%	3.4E-45	ATt	I=I		
C	"	5900	1	T -> C	SNP (transition)	18.3%	3.1E-45	GTt	V=V		
G	TGB1	6236	1	A -> G	SNP (transition)	29.5%	5.7E-67	CaA	Q	CgA	R
G	TGB1	6366	1	A -> G	SNP (transition)	13.7%	7.7E-36	AGa	R=R		
A	TGB3	7145	1	T -> A	SNP (transversion)	11.1%	3.1E-10	AtT	I	AaT	N
A	CP	7318	1	T -> A	SNP (transversion)	11.5%	1.7E-14	GCt	A=A		

Supplementary Figure S1. Graphic representation of InterPro and CCD protein domain searches and annotations, including summary tables described in the genome of jasmine virus C Bari isolate. (A) ORF 1, Replicase domains. (B) ORF 2, Triple Gene Block 1. (C) ORF 3, Triple Gene Block 2. (D) ORF 4, Triple Gene Block 3. (E) ORF 5, Coat Protein. (F) ORF 6, Nucleic Acid Binding protein.

(A) ORF 1, Replicase domains.

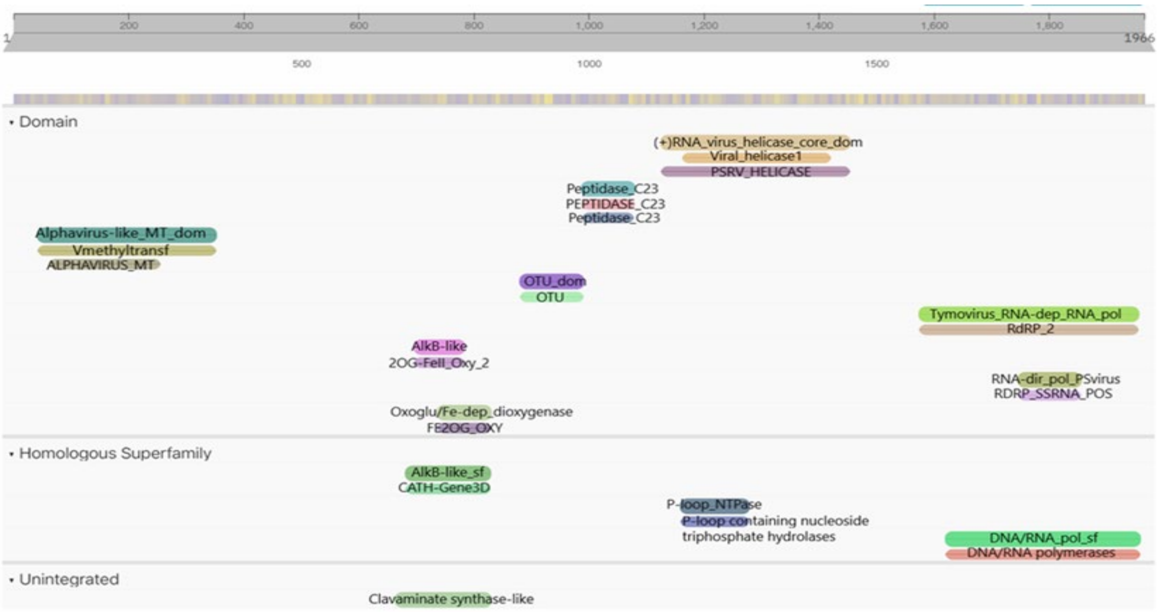

InterPro Name	InterPro ID	InterPro Type	Database	Database ID	Start	End	Length
Alphaviruslike_MT_dom	IPR002588	Domain			43	351	308
Vmethyltransf			Pfam	PF01660	43	351	308
ALPHAVIRUS_MT			PROSITE	PS51743	63	254	191
AlkB-like_sf	IPR037151	homologous superfamily			681	828	147
2OG-FeII_Oxy_2			Pfam	PF13532	698	783	85
CATH-Gene3D				G3DSA:2.60.120.590	681	828	147
Clavaminate synthase-like		Unintegrated	SUPERFA-MILY	SSF51197	690	828	138
Oxoglu/Fe-dep_dioxygenase	IPR005123	Domain			738	829	91
FE2OG_OXY			PROSITE	PS51471	738	829	91
OTU_dom	IPR003323	Domain			882	990	108
OTU			PROSITE	OTU	882	990	108
Peptidase_C23	IPR008041	Domain			989	1079	90
Peptidase_C23			PROSITE	PS51492	989	1079	90
Peptidase_C23			Pfam	PF05379	990	1077	87
(+)RNA_virus_helicase_core_dom	IPR027351	Domain			1138	1460	322
Viral_helicase1			Pfam	PF01443	1164	1420	256
PSRV_HELICASE			PROSITE	PS51657	1138	1460	322
Tymovirus_RNAdep_RNApol	IPR001788	Domain			1576	1955	379
RdRP_2			Pfam	PF00978	1576	1955	379
RNA-dir_pol_PSvirus	IPR007094	Domain			1748	1955	207
RDRP_SSRNA_POS			PROSITE	PS50507	1748	1955	207

(B) ORF 2, Triple Gene Block 1.

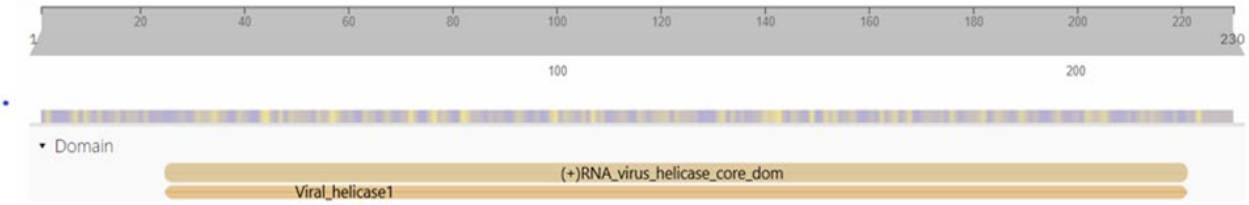

InterPro Name	InterPro ID	InterPro Type	Database	Database ID	Start	End	Length
(+)RNA_virus_helicase_core_dom	IPR027351	Domain			25	221	196
Viral_helicase1			Pfam	PF01443	25	221	196

(C) ORF 3, Triple Gene Block 2.

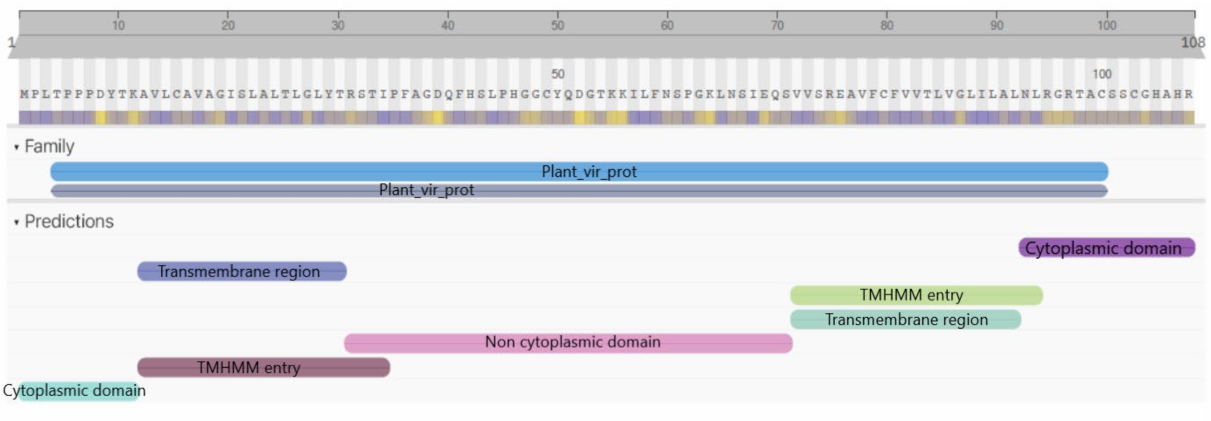

InterPro Name	InterPro ID	InterPro Type	Database	Database ID	Start	End	Length
Cytoplasmic domain			PHOBIUS	CYTOPLASMIC_DOMAIN	1	11	11
Plant_vir_prot	IPR001896	Family			4	99	95
Plant_vir_prot			Pfam	PF01307	4	99	95
Transmembrane region			PHOBIUS	TRANSMEMBRANE	12	30	18
Non cytoplasmic domain			PHOBIUS	NON_CYTOPLASMIC_DOMAIN	31	71	40
TMHMM			TMHMM	TMhelix	72	94	22
Transmembrane region			PHOBIUS	TRANSMEMBRANE	72	92	20
Cytoplasmic domain			PHOBIUS	CYTOPLASMIC_DOMAIN	93	108	15

(D) ORF 4, Triple Gene Block 3.

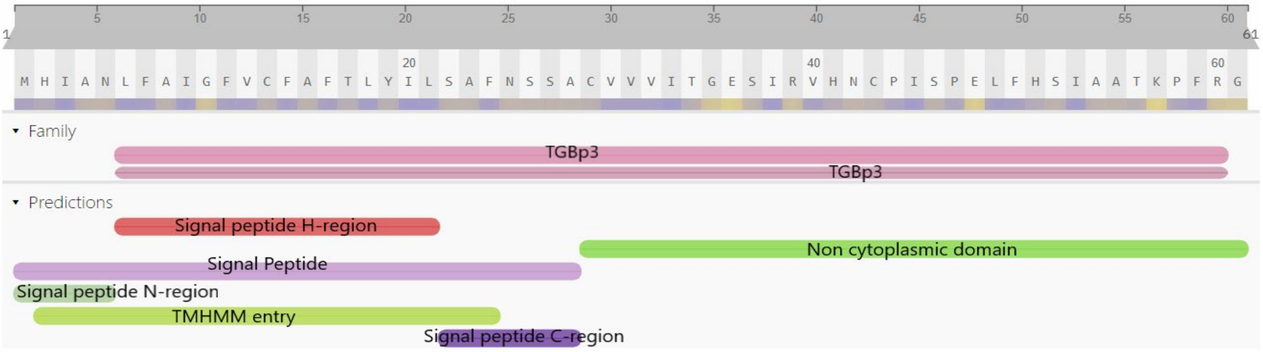

InterPro Name	InterPro ID	InterPro Type	Database	Database ID	Start	End	Length
TGBp3	IPR003411	Family			6	60	54
TGBp3	PF02495		Pfam		6	60	54
Signal peptide H-region			PHOBIUS	SIGNAL_PEPTIDE_H_REGION	6	21	15
Signal Peptide			PHOBIUS	SIGNAL_PEPTIDE	1	28	28
Signal peptide N-region			PHOBIUS	SIGNAL_PEPTIDE_N_REGION	1	5	5
Signal peptide C-region			PHOBIUS	SIGNAL_PEPTIDE_C_REGION	22	28	6
TMHMM			TMhelix	TMHMM	2	24	22
Non cytoplasmic domain			PHOBIUS	NON_CYTOPLASMIC_DOMAIN	29	61	32

(E) ORF 5, Coat Protein.

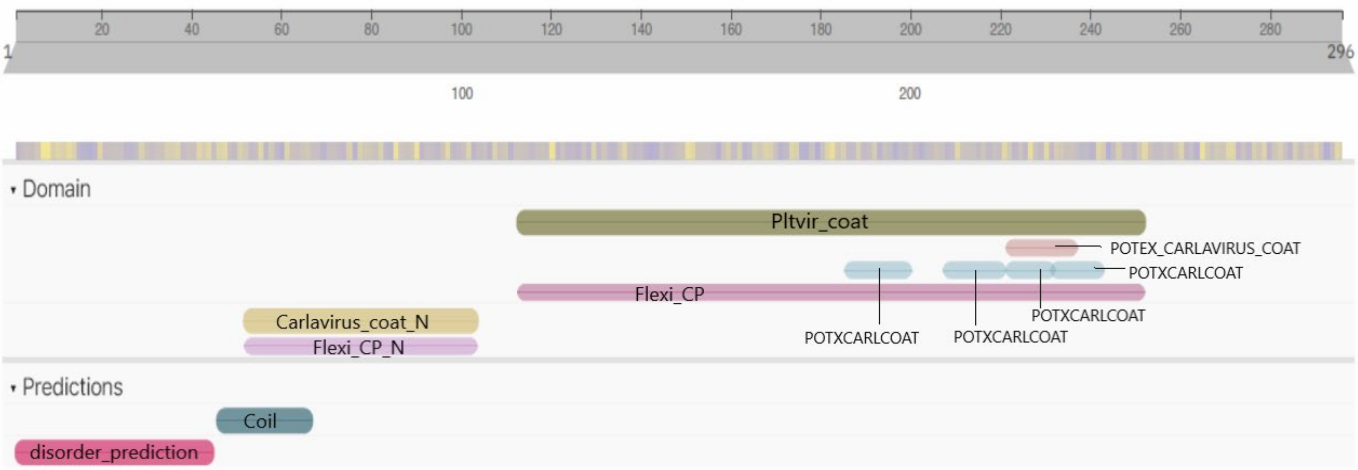

InterPro Name	InterPro ID	InterPro Type	Database	Database ID	Start	End	Length
Disorder_prediction			MOBIDB_LITE	mobidb-lite	1	42	42
Coil	Unintegrated		COILS entry	Coil	46	66	20
Carlavirus_coat_N	IPR013569	Domain			52	103	51
Flexi_CP_N			Pfam	PF08358	52	103	51
Pltvir_coat	IPR000052	Domain			112	252	140
Flexi_CP			Pfam	PF00286	112	252	240
POTXCARLCOAT			PRINTS	PR00232	186	200	14
POTXCARLCOAT			PRINTS	PR00232	208	221	13
POTXCARLCOAT			PRINTS	PR00232	222	232	10
POTXCARLCOAT			PRINTS	PR00232	232	243	11

(F) ORF 6, Nucleic Acid Binding Protein.

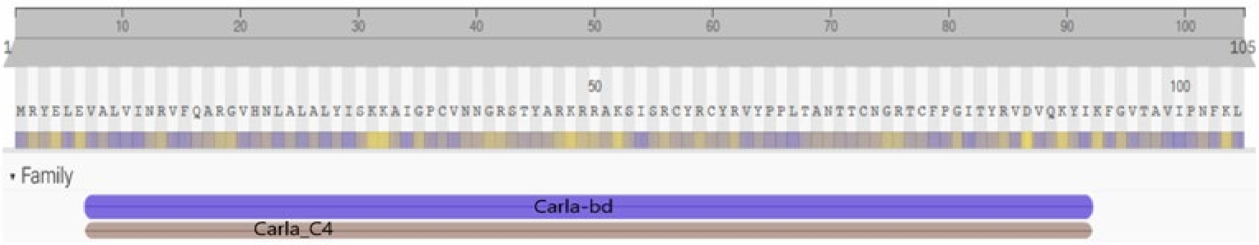

InterPro Name	InterPro ID	InterPro Type	Database	Database ID	Start	End	Length
Carla-bd	IPR002568	Family			7	92	85
Carla_C4			Pfam	PF01623	7	92	85

Supplementary Figure S2.

Alignment (obtained with Clustal Omega; https: //www.ebi.ac.uk/Tools/msa/clustalo/) of the nucleic acid binding proteins (ORF6 protein) of JaVC available in GenBank. Amino acid residues of the Bari isolate (from *J. officinale*) that diverge in the position from the almost invariant residues aligned from all the other isolates (from *J. sambac*) are in red. Accession numbers: Yunnan, MH389795; A31 Taiwan, KX364696; Fujian, MH231174; Guangdong, MH389788; Guangxi, MH389789; Hunan, MH389790; Jilin, MH389791; Sichuan, MH389793; Jangsu, MH389792; Shandong, MH389794.

```
Bari           MRYELEVALVINRVFQARGVHNLALALYISKKAIGPCVNNGRSTYARKRRAKSISRCYRC  60
Guangxi        MRYELEVALIINRVFQARGVHNLALALYISKKAAGPCLNNGRSTYARRRRARSISRCYRC  60
Shandong       MRYELEVALVINRVFQARGVHNLALALYISKKAVGPCLNNGRSTYARRRRARSISRCYRC  60
Hunan          MRYELEVALVINRVFQARGVHNLALALYISKKAVGPCLNNGRSTYARRRRARSISRCYRC  60
Jangsu         MRYELEVALVINRVFQARGVHNLALALYISKKAVGPCLNNGRSTYARRRRARSISRCYRC  60
Jilin          MRYELEVALVINRAFQARGVHNLALALYISKKAVGPCVNKGRSTYARRRRATSISRCYRC  60
Fujian         MRYELEVALVINRAFQARGVHNLALALYISKKAVGPYVNNGRSTYARKRRARSISRCYRC  60
Sichuan        MRHELEVALAINRVFQARGVHNLALALYISKKAVGPCVNNGRSTYARRRRASSISRCYRC  60
Yunnan         MRYELEVALVINRVFQARGVHDLALALYISKKAVGPCVNNGRSTYARKRRAKSISRCYRC  60
A31            MRYELEVALVINRVFQARGVHNLALALYISKKAVGPCVNNGRSTYARRRRAKSISRCYRC  60
Guangdong      MRYELEVALVINRVFQARGVHNLALALYISKKAVGPCVNNGRSTYARRRRAKSISRCYRC  60
               **:****** ***.*******:*********** ** :*:*******:*** ********

Bari           YRVYPPLTANTTCNGRTCFPGITYRVDVQKYIKFGVTAVIPNFKL                105
Guangxi        YRVYPPLSGNTRCDGRTCFPGINYRVDVEEYIKFGVAAAIPNFKL                105
Shandong       YRVYPPLSGNTRCNGRTCFPGINYRVDVEEYIKFGVAAAIPNFEL                105
Hunan          YRVYPPLSGNTRCDGRTCFPGINYRVDVEEYIKFGVAAAIPNFEL                105
Jangsu         YRVYPPLSGNTRCDGRTCFPGINYRVDVEEYIKFGVAAAIPNFKL                105
Jilin          YRVYPPLCGNTRCNGRTCFPGINYRVDVEEYIKFGVAAAIPNFKL                105
Fujian         YRVYPPLCGNTRCNGRTCFPGINYRVDVEKYIKFGVAAAIPNFKL                105
Sichuan        YRVYPPLCGNTRCNGRTCFPGINYRVDVEKYIKFGVAAAIPNFKL                105
Yunnan         YRVYPPLCGNTRCNGRTCFPGINYRVDVEKYIKFGVAAAIPNFKL                105
A31            YRVYPPLCGNTRCNGRTCFPGINYRVDVEKYIKFGVAAAIPNFKL                105
Guangdong      YRVYPPLCGNTRCNGRTCFPGINYRVDVEKYIKFGVAAAIPNFKL                105
               ******* .** *:********.*****::******:*.****:*
```

Supplementary Figure S3.

Alignment (obtained with Clustal Omega; https://www.ebi.ac.uk/Tools/msa/clustalo/) of coat proteins of selected Carlavirus members used to calculate the pairwise percentage similarity among aphid-transmitted and whitefly-transmitted species. Melon: melon yellowing-associated virus, MYaV, NC_038324; cowpea: cowpea mild mottle virus, CPPMV; carya : Carya illinoiensis carlavirus 1, MW328759; narcissus: Narcissus common latent virus, NCLV, NC_008266; A-31: jasmine virus C, Taiwan isolate A-31, KX364696; carnation: carnation latent virus, CLV, MN450069; shallot: shallot latent virus, SLV, NC_003557.

```
melon          MFSGESNDHFSSRLKKYNEVRKALVSQGKTPEEVKEELDEQVERDRKVFEGRQLAGDTGG  60
cowpea         -----------------------MESVFDLNKLVDSEMAEK----TK-------------  20
carya          -----------------------------MPQKTESELAAE----AAFAKTAEEA---K-  23
narcissus      -----------------------MSGAFGVDTVSESSLAAQ----KAL--DRAEA-----  26
JaVC_A-31      -----------------------MS------STSDDSSKGK----RPVVIPETQT-----  22
JaVC_Bari      -----------------------MS------STSDDDTKGK----KPVVIPETQT-----  22
carnation      -----------------------------MPEKRLEEDV---------------------  10
shallot        -----------------------------MT--NEEEEL---------------------  8
                                                  ..

melon          SVVMATEKTEQKTSNPDAKDKGQSSNSKGKVE--EIK----AAQGDISDFQIARLEALKE  114
cowpea         -----------------------TS---------VPKAPDGTALPDIDAELQKRLDDLRA  48
carya          -----RRAAEMEAKRREDKG--KDV---------ETGQRSGSQTPEEEDKLEVMLEKLID  67
narcissus      -------------NQKNADK----L---------KKFAQPESDGSDDEASLEQRLNKLRE  60
JaVC_A-31      -------------NQPPPER--PAS---------EHADSHNFEADTQEEQLEVRMQKLRQ  58
JaVC_Bari      -------------SQPPPGR--PAN---------AGADTHNYEADTQEEQLEARMQKLRQ  58
carnation      -----GSQGQTSPNNPPQQHQGEQSVVSGMVSVNEVDLRNLQNRVEEAEKFMQRFQXLKE  65
shallot        -----NRVRNLPTRNPGTELEGERSKAV-----NDVGVMEREGFEAVLRRSEDRFNKLKE  58
                                                                     :: *

melon          WYRANMRPSDVANPLMQSGLKQMEVTDKLKEDSTNLYSRPALNTLLKWNRAPVSQNIATA  174
cowpea         FLRKTQSASEITNPGFELGRPELKQSTFNSDKHTHIYGKWSIDQLSRIVPKKISNNMATA  108
carya          YRRREYRTSRVTNAGYETGRPLLRTPSNMRGDPSNMYNMLSIDDLYAIKPKAVSNNMATA  127
narcissus      FLRKQRGAIRVTNIGFETGRPTLNLADDMRPDPSNPYNRPSIEALATIKPRAVSNNMATT  120
JaVC_A-31      FLKQQQRATQVTNPSFELGRPKLKMLEGVRSDPTNLYNKPTIDQLCAIKPRAISNNMATS  118
JaVC_Bari      FLKQQQRATQVTNPSFELGRPKLKMLDGVRSDPTNLYNKPTIDQLCSIKPRAISSNMATS  118
carnation      FNSQNLTAGELKNGGFESGRPPAKISEHLRGSTANVFTRPSLDALQMMDFKPESNMIVTA  125
shallot        KCIAELSCINVTNCGWESGRPKAQLADSLKGDASNIFTRPSMDALLVRNYAPESNNLATA  118
                         : *   : *    .       . :: :   ::: *        *. :.*:

melon          EDIAQITSKLESMGVPRERVRDAIFDIVLYAAHNSSSPFQTFEGDIDFRDPKFKSTFSRV  234
cowpea         EEMAKVQITLEGLGVPTEHVAEVLLQVAIYCKDVSSSSFMDSSGTFDWKGGSI----LSD  164
carya          EDMAKIKVSLEALGVPTEEVSTLVAQAAIYCKDTSSSEYMDPRGTFNTSAGAI----SMD  183
narcissus      EDMMRIYTDLEGLGVPTEQVQTVVIQAVLFCKDASSSVFLDPRGSFEWIGGAI----SAD  176
JaVC_A-31      QDMAAITVAIEALGVPSEHIQAVLIQAVAYCKDASSSTYLDPQGVFEWGSGAV----MAD  174
JaVC_Bari      QDMAAITVAIEALGVPSERIQTVLIQAVTYCKDASSSTFLDPQGVFEWEGGAV----MAD  174
carnation      EELAAITAKLEGLGVPTERSAPICWAVARYCANTSTSPYTDPKGVFEFPGGAI----TRD  181
shallot        EELAKISAKVQALGAPEECLAEVFWDICMYCTTAGSSPNVNPKGTISIGGRVV----TRD  174
               :::  :   ::.:*.* *           :.   .:*      * :.     .

melon          SVSSII-KDHSTLRKVCRLFAPIVWSYMIINNEPPANWQAKGFPENAKFASFDTFDFVTN  293
cowpea         SVLAALRKDDNTLRRVCRLYAPITWNFMLTHKAPPSDWVAMGFKWDDRYAAFDCFEYVEN  224
carya          AVFAVLKTQGGTLRRVCRLYAAVTWNYMLIHDAPPSDWPAMGFKRNERFAAFDCLDYVEN  243
narcissus      AVIAVLKKDASTLRRVCRLYAPVTWNYMLTHNAPPSDWAAMGFQHEDRFAAFDCFDYVEN  236
JaVC_A-31      SILAILKRDAGTLRRVCRLYAPVTWNHMLAHNSPPSDWAAMGFQYTERFAAFDCFDYVEN  234
JaVC_Bari      SILAILKRDAGTLRRVCRLYAPITWNHMLAHNSPPSDWAAMGFQYTERFAAFDCFDYVEN  234
carnation      AVFAVIR-EITTLRAFCRAFAPITWNQMLFAKSPPENWQAKGYTHETRYAAFDVFDFVQN  240
shallot        MVVAVIK-EYSTLRQVCRCYAPVVWNYMLLNEQPPANWDAKGFTENTKYAAFDTFDAVTN  233
                : : :  :  *** .** :* :.*. *:  . ** :* * *:    ::*:** :: * *

melon          HSAMKPLEGLVRLPTDIEKLAHDVHKTNSLNKARRNEKLLSTSSKISGGQQACEVKGEFK  353
cowpea         PAAIQPAEGLIRKPTSSEKIAHNTYKRLALDRSNRNEIYSNLNTEVTGGTLGPEISRNFN  284
carya          TAAIPPKEGLIRKPTSNEYIAHNTYKRIALDRSNRNEPFGNLQTEITGGRFGEEIERNHL  303
narcissus      SAAVQPLEGLIRRPTPREKIAHDTHKDIALRAANRNQVFGNFSSGVTGGRNGPELTRNYG  296
JaVC_A-31      SAAVQPFEGLIGRPTPAEKIAHNTHKRLALDRANRNEKYASFEAEVTGGHVGPEIERGIW  294
JaVC_Bari      AAAVQPFEGLIRRPTPAEKIAHNTHKRLALDRANRNEKFANLEAEVTGGHVGPEIERSIW  294
carnation      PAAIQPLEGLLRIPTAEEKIAHATNKRLALDRNRRNARFSSTDSLVTGGMYGKDIKTNFN  300
shallot        KAAIQPLEGLIRAPTDAERIAFATHKKLALAKNSQNSRYANTSAEVTGGFFGCVPKNNFR  293
                :*: * ***:  **  * :*. . *  :*    :*    . .: ::**  .
```

melon	GNGSCS	359
cowpea	HAKK--	288
carya	RPNK--	307
narcissus	NSGLR-	301
JaVC_A-31	KR----	296
JaVC_Bari	KR----	296
carnation	GSNNSD	306
shallot	ENRC--	297

Percent Identity Matrix - created by Clustal 2.1

	MYaV	CPPMV	carya	NCLV	A-31	JaVC-Ba	CLV	SLV
1: melon	100.00	32.16	30.13	34.12	30.93	31.96	35.12	38.14
2: cowpea	32.16	100.00	52.48	50.52	51.79	52.50	42.60	36.73
3: carya	30.13	52.48	100.00	54.76	48.30	47.96	38.57	36.43
4: narcissus	34.12	50.52	54.76	100.00	56.16	56.85	39.08	39.15
5: JaVC A-31	30.93	51.79	48.30	56.16	100.00	90.88	39.50	36.92
6: JaVC Bari	31.96	52.50	47.96	56.85	90.88	100.00	38.79	36.20
7: carnation	35.12	42.60	38.57	39.08	39.50	38.79	100.00	44.44
8: shallot	38.14	36.73	36.43	39.15	36.92	36.20	44.44	100.00
